# Guanylate-binding protein 5 licenses caspase-11 for Gasdermin-D mediated host resistance to *Brucella abortus* infection

**DOI:** 10.1371/journal.ppat.1007519

**Published:** 2018-12-27

**Authors:** Daiane M. Cerqueira, Marco Túlio R. Gomes, Alexandre L. N. Silva, Marcella Rungue, Natan R. G. Assis, Erika S. Guimarães, Suellen B. Morais, Petr Broz, Dario S. Zamboni, Sergio C. Oliveira

**Affiliations:** 1 Departamento de Bioquímica e Imunologia, Instituto de Ciências Biológicas, Universidade Federal de Minas Gerais, Belo Horizonte, Minas Gerais, Brazil; 2 Departamento de Biologia Celular, Universidade de São Paulo-Ribeirão Preto, Brazil; 3 Department of Biochemistry, University of Lausanne, Epalinges, Switzerland; 4 Instituto Nacional de Ciência e Tecnologia em Doenças Tropicais (INCT-DT), Conselho Nacional de Desenvolvimento Científico e Tecnológico, Ministério de Ciência Tecnologia e Inovação Salvador, Bahia, Brazil; University of California, Davis, UNITED STATES

## Abstract

Innate immune response against *Brucella abortus* involves activation of Toll-like receptors (TLRs) and NOD-like receptors (NLRs). Among the NLRs involved in the recognition of *B*. *abortus* are NLRP3 and AIM2. Here, we demonstrate that *B*. *abortus* triggers non-canonical inflammasome activation dependent on caspase-11 and gasdermin-D (GSDMD). Additionally, we identify that *Brucella*-LPS is the ligand for caspase-11 activation. Interestingly, we determine that *B*. *abortus* is able to trigger pyroptosis leading to pore formation and cell death, and this process is dependent on caspase-11 and GSDMD but independently of caspase-1 protease activity and NLRP3. Mice lacking either caspase-11 or GSDMD were significantly more susceptible to infection with *B*. *abortus* than caspase-1 knockout or wild-type animals. Additionally, guanylate-binding proteins (GBPs) present in mouse chromosome 3 participate in the recognition of LPS by caspase-11 contributing to non-canonical inflammasome activation as observed by the response of *Gbp*^*chr3-/-*^ BMDMs to bacterial stimulation. We further determined by siRNA knockdown that among the GBPs contained in mouse chromosome 3, GBP5 is the most important for *Brucella* LPS to be recognized by caspase-11 triggering IL-1β secretion and LDH release. Additionally, we observed a reduction in neutrophil, dendritic cell and macrophage influx in spleens of *Casp11*^*-/-*^ and *Gsdmd*^*-/-*^ compared to wild-type mice, indicating that caspase-11 and GSDMD are implicated in the recruitment and activation of immune cells during *Brucella* infection. Finally, depletion of neutrophils renders wild-type mice more susceptible to *Brucella* infection. Taken together, these data suggest that caspase-11/GSDMD-dependent pyroptosis triggered by *B*. *abortus* is important to infection restriction *in vivo* and contributes to immune cell recruitment and activation.

## Author summary

*Brucella abortus* is the causative agent of brucellosis, a zoonotic disease that affects both humans and cattle. In humans, it is characterized by undulant fever and chronic symptoms as arthritis, endocarditis, and meningitis, while in cattle it causes abortion and infertility. Due to its difficult diagnosis and treatment, it leads to severe economic losses and human suffering. Recently, a novel non-canonical inflammasome pathway was described that involves sensing of bacterial LPS by an intracellular receptor termed caspase-11 and leads to pyroptosis and non-canonical NLRP3 inflammasome activation. Here, we show that *B*. *abortus* or its purified LPS is able to activate the non-canonical inflammasome. In this process, activated caspase-11 leads to GSDMD-dependent pyroptosis. Moreover, this pathway was dependent of IFN-induced GBP proteins, mainly GBP5. To analyze the role of caspase-1, caspase-11 and GSDMD in controlling *B*. *abortus* infection, we infected knockout (KO) mice for these molecules and we observed that caspase-11 and GSDMD KO animals were more susceptible to infection compared to wild-type animals. *Casp11*^*-/-*^ and *Gsdmd*^*-/-*^ animals also recruited less immune cells in mouse spleens compared to wild-type animals in response to *B*. *abortus*. Thus, caspase-11 and GSDMD are major components of the innate immune system to restrict *B*. *abortus in vivo*. This pathway of bacterial sensing by the host immune system is important to future development of drugs and vaccines that may contribute to the control of brucellosis worldwide.

## Introduction

Inflammasomes are multiprotein complexes that assemble in response to pathogen- and damage-associated molecular patterns (PAMPs and DAMPs). The NLRP3 inflammasome, via the adaptor molecule ASC, leads to caspase-1 activation and release of proinflammatory cytokines such as IL-1β and IL-18 [1, 2]. An extensive range of stimuli can trigger the canonical activation of this inflammasome such as damage and stress indicative signals [3–5], environmental insults [6–9], microbial products [10, 11] and bacterial pore-forming toxins [12]. However, recent studies have shown that Gram-negative bacteria can trigger the NLRP3 inflammasome in a non-canonical manner, that depends on caspase-11 [13, 14]. In this process, caspase-11, which was shown to be critical during septic shock [15–19], recognizes bacterial LPS in the cytoplasm, dependent on mouse chromosome 3 GBPs [20–22]. More recently, studies unveiled a pyroptosis mechanism in which active caspase-11 cleaves a protein named Gasdermin D (GSDMD) in its C-terminal p20 and N-terminal p30 fragments [23, 24]. The p30 N-terminal domain inserts and oligomerizes into the plasma membrane forming pores with a diameter of 15–20 nm [25, 26]. Osmotic imbalance triggered by membrane pore formation thereby culminates in membrane rupture and cell death termed pyroptosis [27]. Through the membrane pore, products such as IL-1β [28], ions as potassium, eicosanoids and other proinflammatory molecules can be released [27, 29]. Potassium efflux from the cells is one of the mechanisms believed to trigger NLRP3 inflammasome activation leading to caspase-1 activation and proinflammatory cytokine maturation [30–35]. Furthermore, cytokines and eicosanoids released through the pores might contribute to restricting infection as they drive the recruitment of neutrophils to the local of the infection in order to remove pyroptotic macrophages by efferocytosis [29, 36].

*Brucella abortus* is a Gram-negative facultative intracellular bacterium that causes in humans and cattle a disease termed brucellosis. In humans, it causes pathological manifestations such as arthritis, endocarditis, and meningitis, while in cattle it leads to abortion and infertility, resulting in serious economic losses to the livestock industry [37]. This pathogen infects primarily antigen-presenting cells (APCs), such as dendritic cells and macrophages [38, 39]. These phagocytes act both as an initial replicative niche as well as vehicles for the systemic dissemination of this pathogen, which will then infect myeloid lineage as liver and spleen macrophages, besides remaining in granulomatous lesions [40]. Once inside host cells, *B*. *abortus* develop an intracellular sophisticated replicative cycle [39]. It delivers effector proteins into macrophages cytoplasm through the *virB* type IV secretion system in order to subvert the normal intracellular traffic and establish a replicative niche inside phagocytes termed rBCV (replicative *Brucella* containing vacuole) [41–43].

The innate immune response against *B*. *abortus* begins upon interaction with APCs through recognition by pattern recognition receptors such as TLRs and NLRs [44]. MyD88 and IRAK4 are critical molecules involved in TLRs signaling pathway which results in the activation of NF-κB, MAPKs and production of inflammatory cytokines. These molecules play an essential role for production of proinflammatory cytokines by macrophages and control of *B*. *abortus* infection in mice [45–47]. Although *B*. *abortus* modified LPS is a weak activator of TLR4, unlipidated outer membrane protein (OMP) 16 (U-OMP16) derived from *B*. *abortus* is able to trigger TLR4-dependent inflammatory cytokine production [45, 48]. Furthermore, L-Omp19 triggers TLR2-dependent TNF-α and IL-6 production in mouse peritoneal macrophages [49]. While TLR2 and TLR4 play no role controlling *B*. *abortus* infection in mice, TLR9 correlated to restricting infection, and recently TLR9 was shown to be activated by *B*. *abortus* DNA-derived CpG motifs [45, 50]. Previously published studies from our group revealed that *B*. *abortus* can also be recognized by NLR proteins. NOD1 and NOD2 contribute to NLR signaling in response to *B*. *abortus* as NOD1- and NOD2- deficient BMDMs produced reduced levels of TNF-α [51]. Nevertheless, the absence of these molecules was not critical to control *B*. *abortus* infection [51]. Additionally, *B*. *abortus* can trigger activation of ASC-dependent inflammasomes such as NLRP3 and AIM2, leading to caspase-1 activation and IL-1β secretion [44, 52, 53]. In this study, we demonstrated that *Brucella* LPS is sensed by caspase-11 and triggers GSDMD-dependent pyroptosis leading to control of bacterial infection *in vivo*.

## Results

### IL-1β secretion and caspase-1 activation in response to *B*. *abortus* are dependent on NLRP3, caspase-11 and the bacterial type IV secretion system

**Fig 1:**
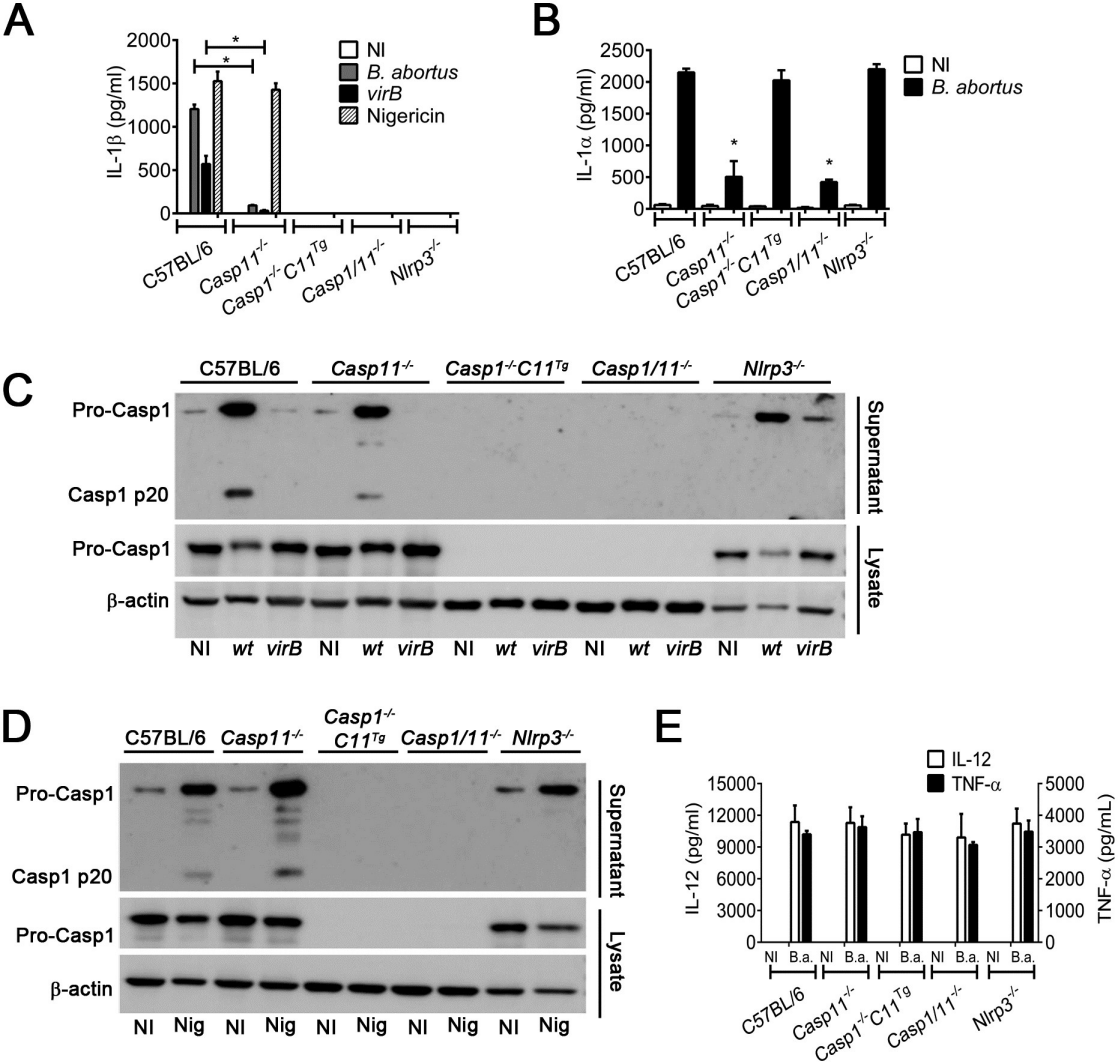
Caspase-11, NLRP3 and type IV secretion system are critical to IL-1β secretion and caspase-1 activation in response to *B*. *abortus*. BMDMs obtained from C57BL/6, *Casp11*^−/−^, *Casp1*^*−/−*^*Casp11*^*Tg*^, *Nlrp3*^*−/−*^ and *Casp1/11*^*−/−*^ mice were primed with *E*. *coli* LPS (1 μg/ml) for 4 h and then left uninfected (NI) or stimulated with *B*. *abortus* or mutant *virB2*. BMDMs were infected with *B*. *abortus* or mutant *virB2* at an MOI of 100 for 17 h of infection. As positive control, BMDMs were stimulated with 20μM nigericin for 50 minutes. The concentration of IL-1β (A) or IL-1α (B) in the culture supernatants was estimated by ELISA. The same culture supernatants and lysates harvested 17 h postinfection were separated by SDS-PAGE, blotted and probed with monoclonal anti-caspase-1 p20 subunit antibody (C). Equal loading was controlled by measuring β-actin in the corresponding cell lysates. As positive control to western blotting, BMDMs were stimulated with 20μM nigericin for 50 minutes (D). Supernatant and cell lysates were separated by SDS-PAGE, blotted, and probed with monoclonal anti-caspase-1 p20 subunit antibody. As loading control, cell lysates were probed with monoclonal anti-β-actin antibody. Macrophage culture supernatants were harvested 17 hrs after infection to also measure IL-12 and TNF-α by ELISA (E). Data show the mean ± SD from triplicate wells. *p < 0.05 compared to C57BL/6. The graphs are representative of three independent experiments. NI: uninfected; *wt*: *B*. *abortus* wild-type; *virB2*: mutant *B*. *abortus*; Nig: nigericin.

Previously, we demonstrated that *B*. *abortus* infects macrophages leading to caspase-1 activation and IL-1β secretion dependent on NLRP3 [52]. Recently, Kayagaki and collaborators described a non-canonical NLRP3 inflammasome activation pathway dependent on caspase-11 [13]. However, the role of caspase-11 during *B*. *abortus* infection was still unknown. Thus, we investigated whether NLRP3 inflammasome activation in response to *B*. *abortus* required caspase-11. We infected LPS-primed C57BL/6, *Casp11*^*-/-*^, *Nlrp3*^*-/-*^ and *Casp1/11*^*-/-*^ BMDMs with *B*. *abortus* and measured IL-1β secretion and caspase-1 cleavage. We also infected BMDM from *Casp1/11*^*−/−*^ mice expressing a functional caspase-11 allele to generate single caspase-1-deficient mice (hereafter termed *Casp1*^*−/−*^*Casp-11*^*Tg*^) [13]. After 17 hours of infection, secretion of IL-1β into the supernatant was evaluated. We observed that *Casp11*-, *Nlrp3*- and *Casp1*-single-deficient BMDMs reduced the levels of IL-1β released in comparison to C57BL/6 (Fig 1A). The remaining IL-1β release observed in *Casp11*-deficient macrophages is probably due to canonical NLRP3 inflammasome activation. As expected, non-infected macrophages did not release significant levels of IL-1β. To evaluate the importance of the *B*. *abortus* type IV secretion system for IL-1β secretion, we also infected these macrophages with type IV secretion system deficient *B*. *abortus* (Δ*virB2*) and observed that all macrophages secreted reduced levels of IL-1β in response to *B*. *abortus* Δ*virB2* in comparison to WT *B*. *abortus*. As a control, these macrophages were treated with nigericin, a canonical NLRP3 agonist. As expected, we observed that primed C57BL/6 and *Casp11*-deficient BMDMs secreted similar levels of IL-1β, whereas primed *Casp1*^*-/-*^*Casp11*^*Tg*^, *Casp1/11*^*-/-*^ and *Nlrp3*^*-/-*^ were unable to secrete IL-1β in response to nigericin (Fig 1A). We also decided to investigate whether IL-1α release induced by *B*. *abortus* required caspase-11. We infected BMDMs from C57BL/6, *Casp11*^*-/-*^, *Casp1*^*-/-*^*Casp11*^*Tg*^, *Nlrp3*^*-/-*^ and *Casp1/11*^*-/-*^ with *B*. *abortus* and after 17 hours of infection, we collected supernatant and measured IL-1α release. We observed that C57BL/6 and *Casp1*^*-/-*^*Casp11*^*Tg*^ and *Nlrp3*^*-/-*^ secreted similar levels of IL-1α (Fig 1B). However, BMDMs from *Casp11*^*-/-*^ and *Casp1/11*^*-/-*^ released reduced levels of this cytokine suggesting the importance of caspase-11 but not caspase-1 promoting IL-1α release in response to *B*. *abortus*. As expected, non-infected controls did not secrete significant levels of this cytokine.

Next, we assessed if caspase-11 is required for caspase-1 cleavage. We infected primed C57BL/6, *Casp11*^*-/-*^ and *Nlrp3*^*-/-*^ BMDMs with *B*. *abortus*. After 17 hours of infection, cell supernatants were collected and subjected to Western blotting using a specific Ab against the p20 subunit of caspase-1. We observed that *Casp11*-deficient BMDMs showed reduced levels of caspase-1 activation in comparison to C57BL/6 macrophages which were fully able to activate caspase-1 (Fig 1C). The minor caspase-1 processing observed in Casp11-deficient macrophages is probably due to canonical NLRP3 inflammasome activation. As a control, we infected *Casp1*^*-/-*^*Casp11*^*Tg*^ and *Casp1/11*^*-/-*^ which did not express caspase-1. These macrophages were also infected with the *B*. *abortus* type IV secretion system mutant Δ*virB2* and they were not able to activate caspase-1. As a control for cell viability and the ability to cleave caspase-1 in response to a known stimulus, macrophages were treated with nigericin. We observed that C57BL/6 and *Casp11*^*-/-*^ were fully able to activate caspase-1, as expected (Fig 1D); however, *Nlrp3*^*-/-*^ BMDMs were unable to activate caspase-1 and *Casp1*^*-/-*^*Casp11*^*Tg*^ and *Casp1/11*^*-/-*^ did not express caspase-1. In order to assess whether these BMDMs properly express caspase-11, we infected primed C57BL/6, *Casp11*^*-/-*^, *Casp11*^*-/-*^*Casp11*^*Tg*^ and *Casp1/11*^*-/-*^ BMDMs with *B*. *abortus* and analyzed caspase-11 expression. Caspase-11 was efficiently upregulated in response to infection with *B*. *abortus* in C57BL/6 and *Casp1*^*-/-*^*Casp11*^*Tg*^ BMDMs. As expected, BMDMs from *Casp11*^*-/-*^ and *Casp1/11*^*-/-*^ did not express caspase-11 (S1 Fig).

Further, to investigate whether lack of caspase-11 or caspase-1 could interfere in inflammasome-independent cytokines, levels of IL-12 and TNF-α were measured in the supernatants of *Brucella*-infected KO macrophages. As observed in Fig 1E, infected *Casp11*^*-/-*^ and *Casp1/11*^*-/-*^ macrophages produced similar levels of these cytokines when compared to cells of wild-type mice.

Taken together, these data suggest that caspase-11 is required for caspase-1 activation, IL-1β and IL-1α secretion in response to *B*. *abortus*.

### *B*. *abortus* LPS triggers IL-1β secretion and caspase-1 activation dependent on NLRP3 and caspase-11

**Fig 2:**
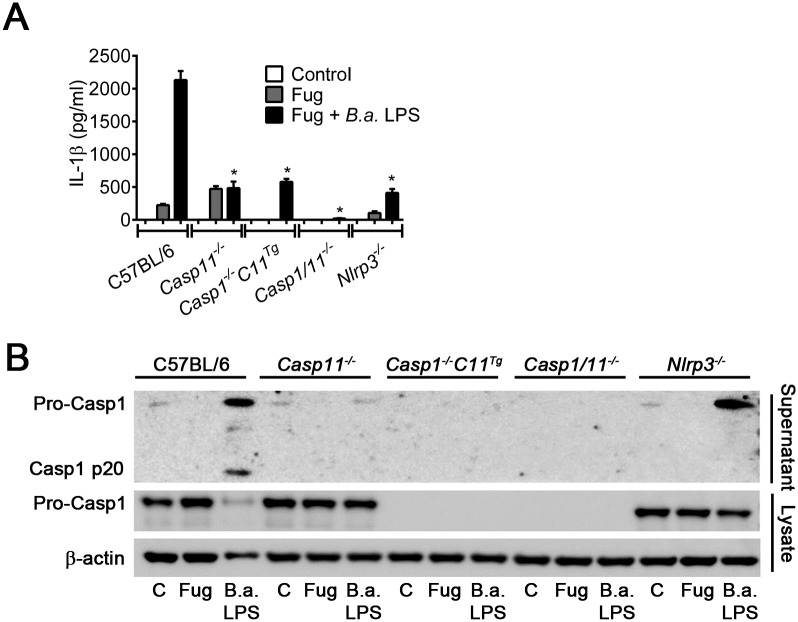
*B*. *abortus* LPS triggers caspase-11 activation and non-canonical inflammasome. BMDMs obtained from C57BL/6, *Nlrp3*^-/-^, *Casp1/11*^-/-^, *Casp1*^*-/-*^*C11*^*Tg*^ and *Casp11*^*-/-*^ mice were previously primed with PAM3CSK (1 μg/ml) for 6 h. Then, they were left untransfected (control) or transfected with *B*. *abortus* LPS (5 μg/ml) using FuGENE transfection reagent according to the manufacturer instructions (Fug + *B*.*a*. LPS). As negative control, BMDMs were treated with FuGENEHD without LPS (Fug). After 17 hrs, the supernatant were harvested. (A) Supernatants were submitted to ELISA to estimate the concentration of IL-1β. (B) Cell supernatants were separated by SDS-PAGE, blotted and probed with an anti-caspase-1 p20 subunit monoclonal antibody. As loading control, cell lysates were probed with anti-β-actin monoclonal antibody. Data show the mean ± SD from triplicate wells. *p < 0.05 compared to C57BL/6. The graphs are representative of three independent experiments.

Previous study suggested that caspase-11 is an intracellular LPS receptor [17]. Once it recognizes LPS in the cytosol, caspase-11 is activated triggering pyroptosis and IL-1α secretion. Moreover, caspase-11 is able to trigger NLRP3/ASC inflammasome activation, leading to caspase-1 processing and IL-1β secretion [15–17]. Thus, we analyzed whether *B*. *abortus* LPS directly transfected into macrophage cytosol was able to trigger caspase-1 activation and IL-1β secretion. BMDMs from C57BL/6, *Nlrp3*^*-/-*^, *Casp1/11*^*-/-*^, *Casp1*^*-/-*^*Casp11*^*Tg*^ and *Casp11*^*-/-*^ mice were transfected with purified *B*. *abortus* LPS and after 17 hours of transfection, we measured IL-β production and caspase-1 activation in the cell supernatant. We observed that C57BL/6 BMDMs were able to produce high levels of IL-1β in response to cytoplasmic LPS (Fig 2A). In contrast, BMDMs from *Casp11*^*-/-*^, *Casp1*^*-/-*^*Casp11*^*Tg*^, *Casp1/11*^*-/-*^ and *Nlrp3*^*-/-*^ mice secreted low levels of IL-1β similar to control cells treated only with transfection reagent FuGENEHD. These data suggested that *B*. *abortus* LPS is recognized by caspase-11 in the macrophage cytosol and leads to IL-1β secretion dependent on NLRP3, caspase-1 and caspase-11.

Also, we investigated whether *B*. *abortus* LPS was able to trigger caspase-1 activation in a caspase-11-dependent manner. BMDMs from C57BL/6, *Nlrp3*^*-/-*^, *Casp1/11*^*-/-*^, *Casp1*^*-/-*^*Casp11*^*Tg*^ and *Casp11*^*-/-*^ mice were transfected with *B*. *abortus* LPS. After 17 hours of transfection, cell supernatants were collected and lysates were prepared for immunoblotting using specific Ab. We observed that wild-type macrophages directly transfected with *B*. *abortus* LPS activates caspase-1 (Fig 2B). In contrast, BMDMs from *Nlrp3*^*-/-*^ and *Casp11*^*-/-*^ mice were not able to activate caspase-1 in response to *B*. *abortus* LPS. As expected, *Casp1*^*-/-*^*Casp11*^*Tg*^ and *Casp1/11*^*-/-*^ BMDMs did not express caspase-1. Moreover, caspase-1 activation was not observed in non-treated and FuGENEHD-treated BMDM controls, as expected. These data suggested that caspase-1 activation in response to *B*. *abortus* LPS is dependent on caspase-11 and NLRP3. Collectively, these data demonstrated that *B*. *abortus* LPS is the PAMP responsible for non-canonical caspase-11 inflammasome activation when recognized by caspase-11 in the macrophage cytosol.

### Macrophage pyroptosis triggered by *B*. *abortus* is dependent on caspase-11 and independent of caspase-1 and NLRP3

**Fig 3:**
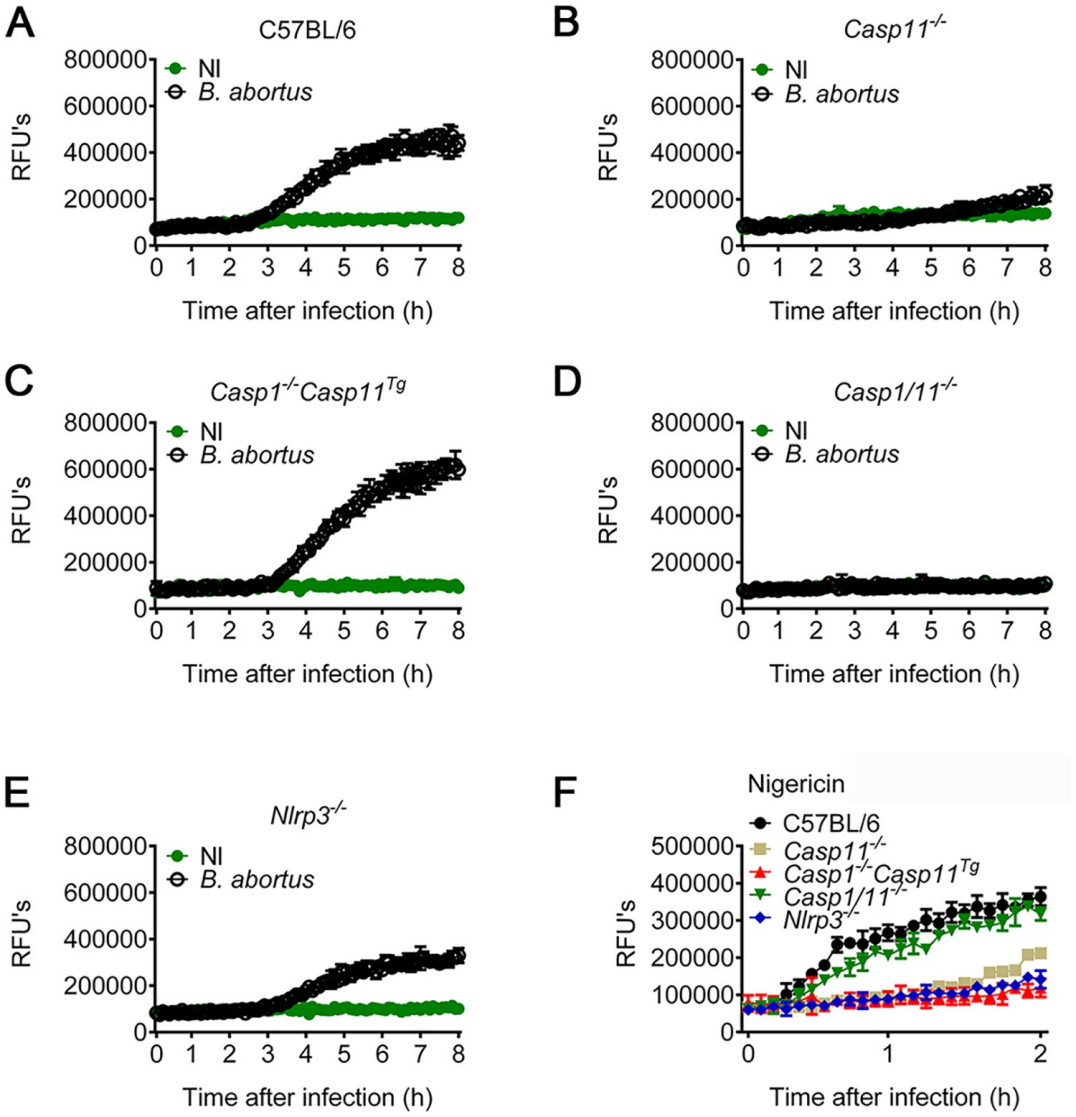
Macrophage pyroptosis in response to *B*. *abortus* requires caspase-11 but not caspase-1. BMDMs obtained from C57BL/6, *Casp11*^−/−^, *Casp1*^*−/−*^*Casp11*^*Tg*^, *Nlrp3*^*−/−*^ and *Casp1/11*^*−/−*^ mice were left uninfected (NI) or stimulated with *B*. *abortus*. (A-E) BMDMs previously primed with *E*. *coli* LPS (1 μg/ml) for 4 h, were left uninfected (●) or infected with *B*. *abortus* (○) at an MOI of 100 for 8 hrs. Shown are pore formation in BMDMs from C57BL/6 (A), *Casp11*^*-/-*^ (B), *Casp1*^*-/-*^*C11*^*Tg*^ (C), *Casp1/11*^*-/-*^ (D) and *Nlrp3*^*-/-*^ (E) mice. As control, BMDMs were treated with 20μM nigericin (F) for 50 min. Pore formation was assessed fluorimetrically in real time by the uptake of propidium iodide (relative fluorescence units) into the nucleus of the permeabilized BMDMs. Data show the mean ± SD from triplicate wells. NI: uninfected; RFU: relative fluorescence unit. The graphs are representative of three independent experiments.

Once caspase-11 is activated, it triggers an inflammatory form of cell death termed pyroptosis, which is independent of NLRP3/caspase-1 axis [13]. Therefore, we asked whether *B*. *abortus* is able to trigger pore formation and pyroptosis. BMDMs from C57BL/6, *Nlrp3*^*-/-*^, *Casp1/11*^*-/-*^, *Casp1*^*-/-*^*Casp11*^*Tg*^ and *Casp11*^*-/-*^ mice were infected with *B*. *abortus* in a medium containing propidium iodide. To assess pore formation, we quantified the influx of propidium iodide into the nuclei of the cells in real time during 8 h of infection. We observed that *B*. *abortus* was able to trigger pore formation in C57BL/6, *Casp1*^*-/-*^*Casp11*^*Tg*^ and *Nlrp3*^*-/-*^ BMDMs, but failed to trigger pore formation in *Casp11*^*-/-*^ and *Casp1/11*^*-/-*^ BMDMs (Fig 3A–3E). Thus, *B*. *abortus* is able to trigger pore formation in macrophages dependent on caspase-11 but independently of caspase-1 or NLRP3. When we stimulated the cells with nigericin as control, *Casp11*-deficient BMDMs were as able to form pores as C57BL/6 BMDMs, whereas *Casp1*^*-/-*^*Casp11*^*Tg*^, *Nlrp3*^*-/-*^ and *Casp1/11*^*-/-*^ failed to trigger pore formation in response to nigericin (Fig 3F). Taken together, these data suggest that *B*. *abortus* triggers pore formation in macrophages dependent on caspase-11 but independent of caspase-1.

### Guanylate-binding proteins (GBPs) are required for macrophage pyroptosis and caspase-11 activation in response to *B*. *abortus*

**Fig 4:**
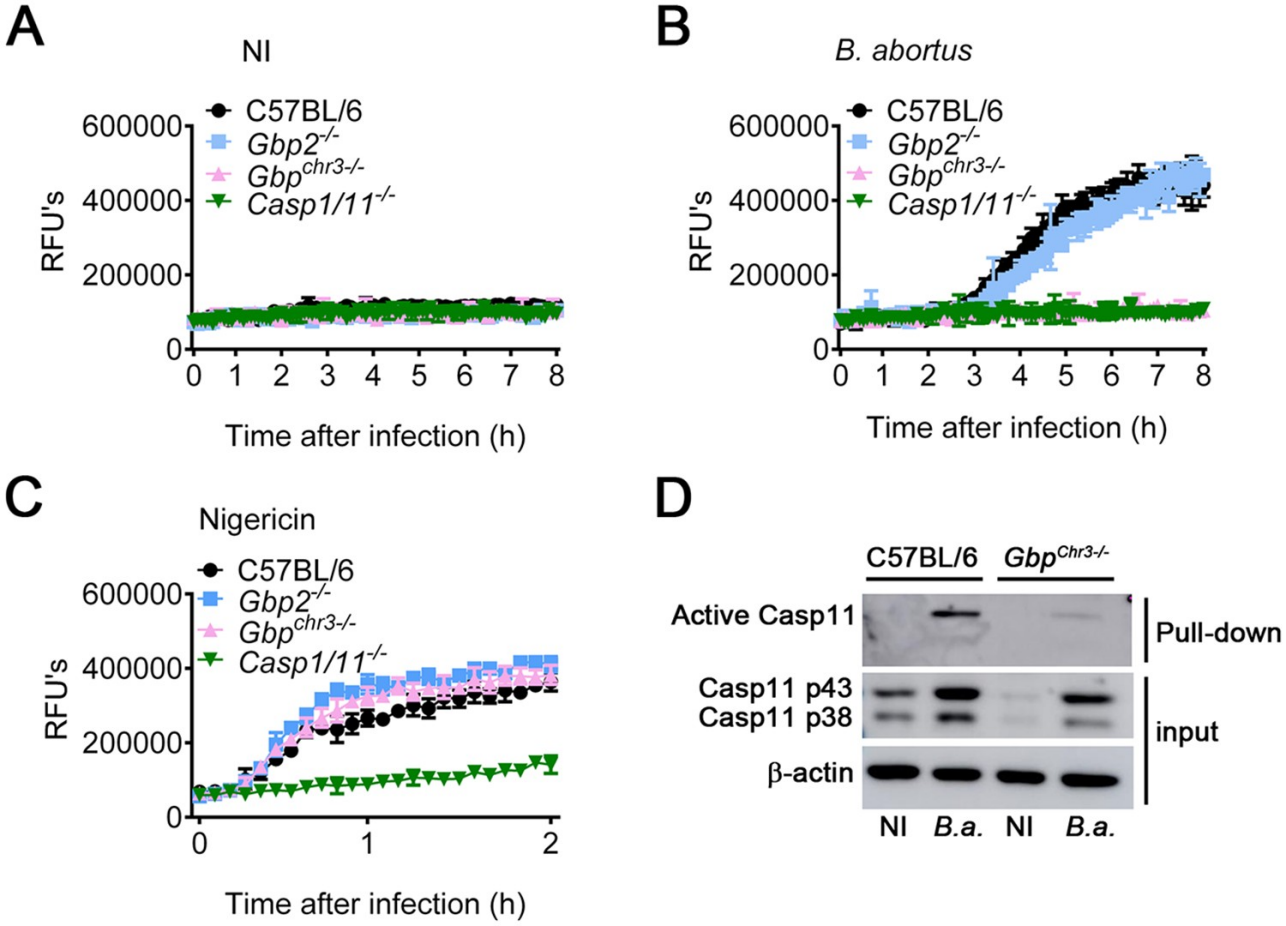
Pyroptosis and caspase-11 activation in response to *B*. *abortus* require guanylate-binding proteins (GBPs). (A-C) BMDMs obtained from C57BL/6, *Gbp2*^-/-^, *Gbp*^*chr3-/-*^ and *Casp1/11*^*-/-*^ were previously primed with *E*. *coli* LPS (1 μg/ml) for 4 h. Then, they were left uninfected (NI) (A), infected with *B*. *abortus* at an MOI of 100 (B) or treated with 20μM nigericin (C). Pore formation was assessed fluorimetrically in real time by the uptake of propidium iodide (relative fluorescence units). (D) Active caspase-11 pulldown assay from the lysates of unprimed C57BL/6 and *Gbp*^*chr3-/-*^ BMDMs. BMDMs were pretreated with biotin-VAD-FMK and after 15 min, they were infected with *B*. *abortus* at an MOI of 100. Pulldown of active caspase-11 bound to biotin-VAD-FMK was performed using agarose-streptavidin beads. Shown are immunoblot of pulldown fraction and total cell lysate (input) using monoclonal caspase-11 antibody. As loading control, total cell lysates were probed with anti-β-actin monoclonal antibody. NI: uninfected; *B*.*a*.: *B*. *abortus*; RFU: relative fluorescence unit. The graphs are representative of two independent experiments.

Guanylate-binding proteins (GBPs) are IFN-inducible GTPases which act both in the membrane disruption of vacuolar pathogens and facilitating intracellular LPS interaction with caspase-11 to activate non-canonical inflammasome, mainly when LPS is within liposomal membranes and within bacterial outer membranes [20–22]. We therefore hypothesized that GBPs participate in the activation of the non-canonical inflammasome by *B*. *abortus*. Thus, we asked whether GBPs are involved in pore-formation in response to *B*. *abortus*. To assess the role of GBPs, we infected BMDMs from C57BL/6, *Gbp*^*chr3-/-*^ (deficient for the locus on mouse chromosome 3 encoding GBP1, GBP2, GBP3, GBP5, and GBP7), *Gbp2*^*-/-*^ and *Casp1/11*^*-/-*^ with *B*. *abortus*. By evaluating propidium iodide uptake in real time during 8 h of infection, we found that *Gbp2*^*-/-*^ BMDMs were able to form pores similar to C57BL/6 BMDMs whereas *Gbp*^*chr3-/-*^ BMDMs failed to form pores in response to *B*. *abortus* as observed for *Casp1/11*^*-/-*^ (Fig 4B). As expected, non-infected controls failed to trigger pore formation (Fig 4A). As a control, we treated BMDMs from C57BL/6, *Gbp*^*chr3-/-*^, *Gbp2*^*-/-*^ and *Casp1/11*^*-/-*^ with nigericin and evaluated propidium iodide uptake in real time during 2 h of treatment. We observed that Gbp2- and Gbp^chr3^-deficient BMDMs form pores at the same level as C57BL/6 BMDMs in response to nigericin whereas *Casp1/11*^*-/-*^ BMDMs failed to replicate this phenotype (Fig 4C). These data suggest that GBP2 seems to be dispensable but other GBPs on mouse chromosome 3 are critical to pore formation in response to *B*. *abortus*. Because caspase-11 activation is required for pore formation, we asked whether GBPs on mouse chromosome 3 are important to caspase-11 activation. Therefore, BMDMs from C57BL/6 and *Gbp*^*chr3-/-*^ mice were pre-treated with a biotin-labeled caspase inhibitor (Biotin-VAD-FMK) which only binds to the active site of activated caspases. After 15 min, cells were infected with *B*. *abortus* for 17 h and subsequently, cell lysates were submitted to pulldown with streptavidin-coupled beads. Then, the pulldown product was subjected to western blotting using specific Ab against caspase-11. We found that in the absence of GBPs from chromosome 3, caspase-11 could not be activated while in wild-type BMDMs caspase-11 was strongly activated (Fig 4D). Altogether, these data suggest that GBPs on mouse chromosome 3 are essential for *Brucella*-driven caspase-11 activation and consequently to non-canonical inflammasome activation.

**Fig 5:**
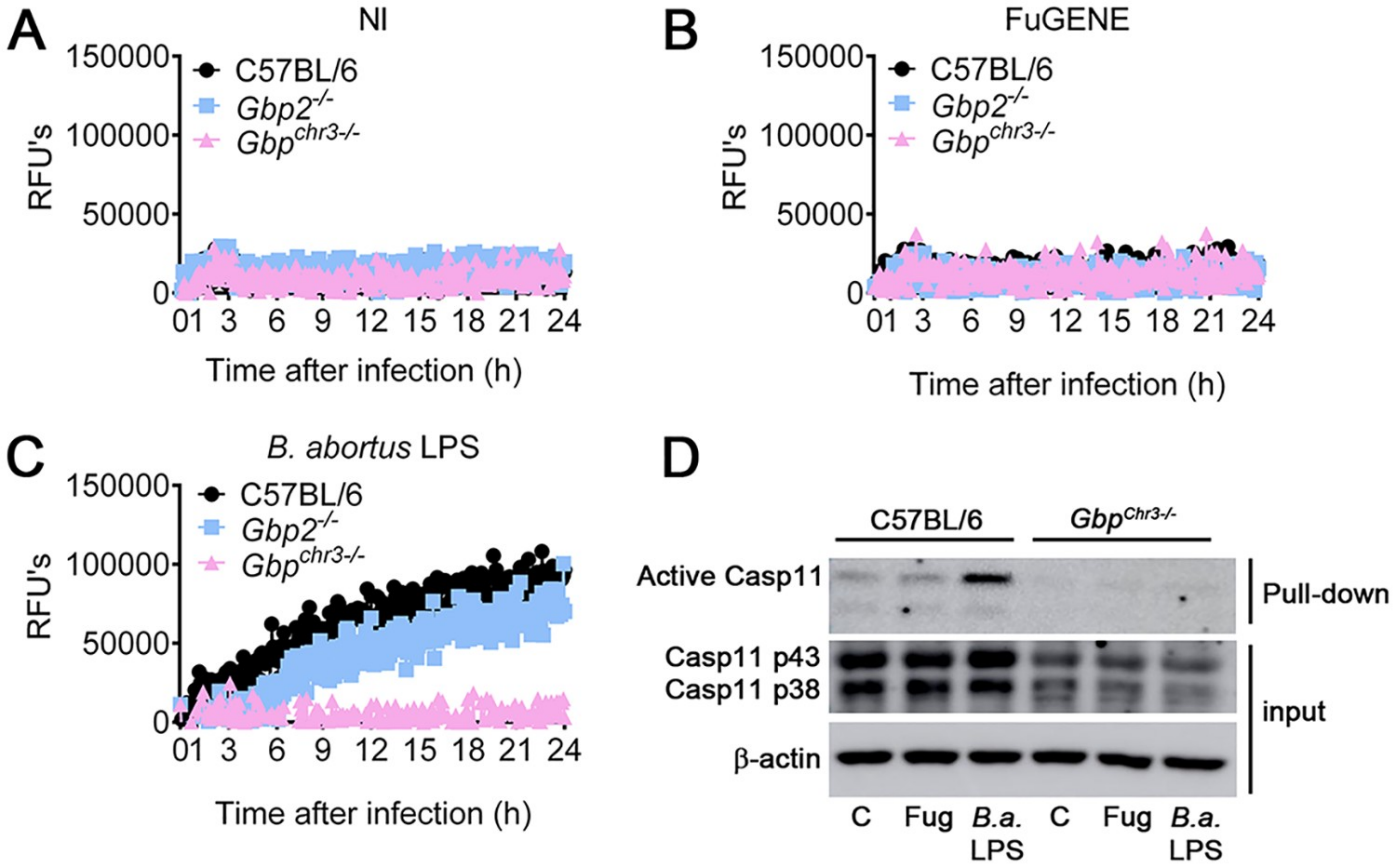
*B*. *abortus* LPS activates caspase-11 and triggers pyroptosis in a guanylate-binding proteins-dependent fashion (GBPs). BMDMs obtained from C57BL/6, *Gbp2*^*-/-*^ and *Gbp*^*chr3-/-*^ pretreated with PAM3CSK (500 ng/ml) for 6 h were left untransfected (NI) (A), treated with FuGENEHD (B) or treated with *B*. *abortus* LPS (5 μg/ml) pre-mixed with FuGENEHD (C) according to the manufacturer's instructions. Pore formation was assessed fluorimetrically in real time by the uptake of propidium iodide (relative fluorescence units). (D) BMDMs were pretreated with biotin-VAD-FMK 15 min before transfection. Cells were transfected with *B*. *abortus* LPS using FuGENEHD for 17 hrs. Immunoblot show the presence of caspase-11 p43 and p38 in the total cell lysate (input) and pull-down fraction using the agarose-streptavidin fraction of BMDMs. β-actin was used as a loading control. NI: untransfected; RFU: relative fluorescence unit; C: control; Fug: treated with FuGENEHD; *B*.*a*. LPS: transfected with *B*.*a*. LPS. The graphs are representative of two independent experiments.

Our data suggest that *B*. *abortus* LPS is the PAMP responsible to activate the non-canonical pathway. Previous studies suggested that caspase-11 acts as an intracellular LPS receptor [17]. However, recent study demonstrated that GBPs have a notable function mediating LPS interaction with caspase-11 [20]. Hence, we asked whether GBPs are important to activation of the non-canonical caspase-11 inflammasome also in response to purified *B*. *abortus* LPS. To test that, we transfected BMDMs primed with PAM3CSK from C57BL/6, *Gbp2*^*-/-*^ and *Gbp*^*chr3-/-*^ mice with *B*. *abortus* LPS using FuGENEHD and evaluated propidium iodide uptake in real time during 8 h of infection. We observed that C57BL/6 and *Gbp2*^*-/-*^ BMDMs were able to form pores in response to *B*. *abortus* LPS whereas *Gbp*^*chr3-/-*^ BMDM failed to form pores in response to bacterial LPS (Fig 5A–5C). Moreover, we investigated whether GBPs on mouse chromosome 3 were crucial to caspase-11 activation also in response to *B*. *abortus* LPS. We previously primed C57BL/6 and *Gbp*^*chr3-/-*^ BMDMs with PAM3CSK during 6 hours. Then, we pretreated these BMDMs with biotin-labeled caspase inhibitor (Biotin-VAD-FMK) and after 15 min transfected them with *B*. *abortus* LPS. After 17 h, cells lysates were submitted to pulldown with streptavidin-coupled beads. To observe caspase-11 activation levels, the pulldown product was subjected to western blotting using specific Ab against caspase-11. As we previously observed to whole bacteria, C57BL/6 BMDMs were able to activate caspase-11 in response to purified *B*. *abortus* LPS whereas Gbp^chr3^-deficient BMDMs failed to activate caspase-11 (Fig 5D).

**Fig 6:**
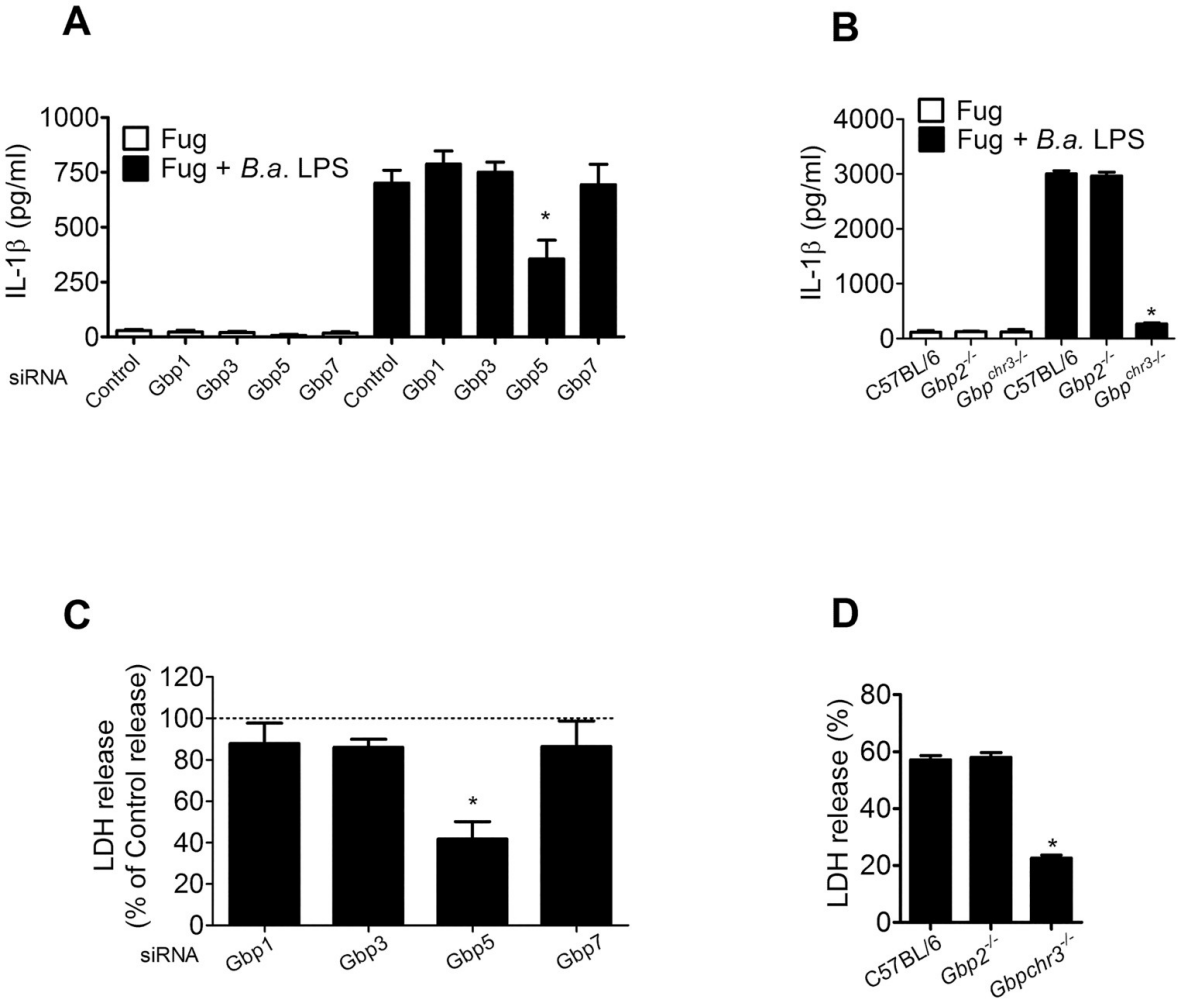
GBP5 is required for IL-1β secretion and LDH release in response to *B*. *abortus* LPS. BMDMs from C57BL/6 mice pretreated with PAM3CSK (500 ng/ml) were transfected with siRNA for GBP1, 3, 5 and 7 for 46 h. Macrophages treated with siRNA or obtained from *Gbp2*^*-/-*^ or *Gbp*^*chr3-/-*^ mice were transfected with FuGENEHD alone or with *B*. *abortus* LPS (5 μg/ml) pre-mixed with FuGENEHD according to the manufacturer's instructions. After 17 h, the supernatants were harvested. (A-B) IL-1β secretion was measured by ELISA. *p < 0.05 compared to control siRNA or C57BL/6. Pyroptosis was assessed by measuring LDH release in the supernatant. (C) For GBP knocked down BMDMs, values represent the percentage of the mean value of LDH release compared to cells transfected with control siRNA which was used as a reference value set to 100%. *p < 0.05 compared to each individual GBP siRNA treated cells transfected with *B*. *abortus* LPS. (D) For knockout BMDMs, values represent the percentage of LDH released compared to cells lysed with Triton X-100. *p < 0.05 compared to C57BL/6 cell cultures transfected with *B*. *abortus* LPS. Data show the mean ± SD representative of two independent experiments.

To investigate which GBPs contained on mouse chromosome 3 (GBP^chr3^) would be involved in LPS sensing by caspase-11, we first performed qPCR analysis of *GBP1*, *GBP2*, *GBP3*, *GBP5* and *GBP7* expression on macrophages transfected with *Brucella* LPS. We observed that *GBP2*, *GBP3*, *GBP5* and to less extent GBP7 had increased mRNA transcripts in macrophages transfected with bacterial LPS compared to cells transfected with FuGENEHD alone (S2 Fig). We then treated wild-type BMDMs with GBP1, GBP3, GBP5 and GBP7 siRNA and transfected them with *B*. *abortus* LPS and measured IL-1β and LDH release. As shown in Fig 6A and 6C, only GBP5 siRNA treated cells reduced IL-1β secretion and LDH release when compared to other knockdowned GBPs. Simultaneously, we also performed similar experiments with GBP2 and GBP^chr3^ KO macrophages and these experiments demonstrated that GBP2 plays no role in IL-1β secretion and LDH release as a result of *Brucella* LPS recognition by caspase-11 (Fig 6B and 6D). Collectively, our data suggest that GBPs on mouse chromosome 3, more specifically GBP5, mediates caspase-11 activation and consequently triggers non-canonical inflammasome in response to purified *B*. *abortus* LPS.

### Gasdermin-D is critical to induce pyroptosis and to activate NLRP3 inflammasome in response to *B*. *abortus*

**Fig 7:**
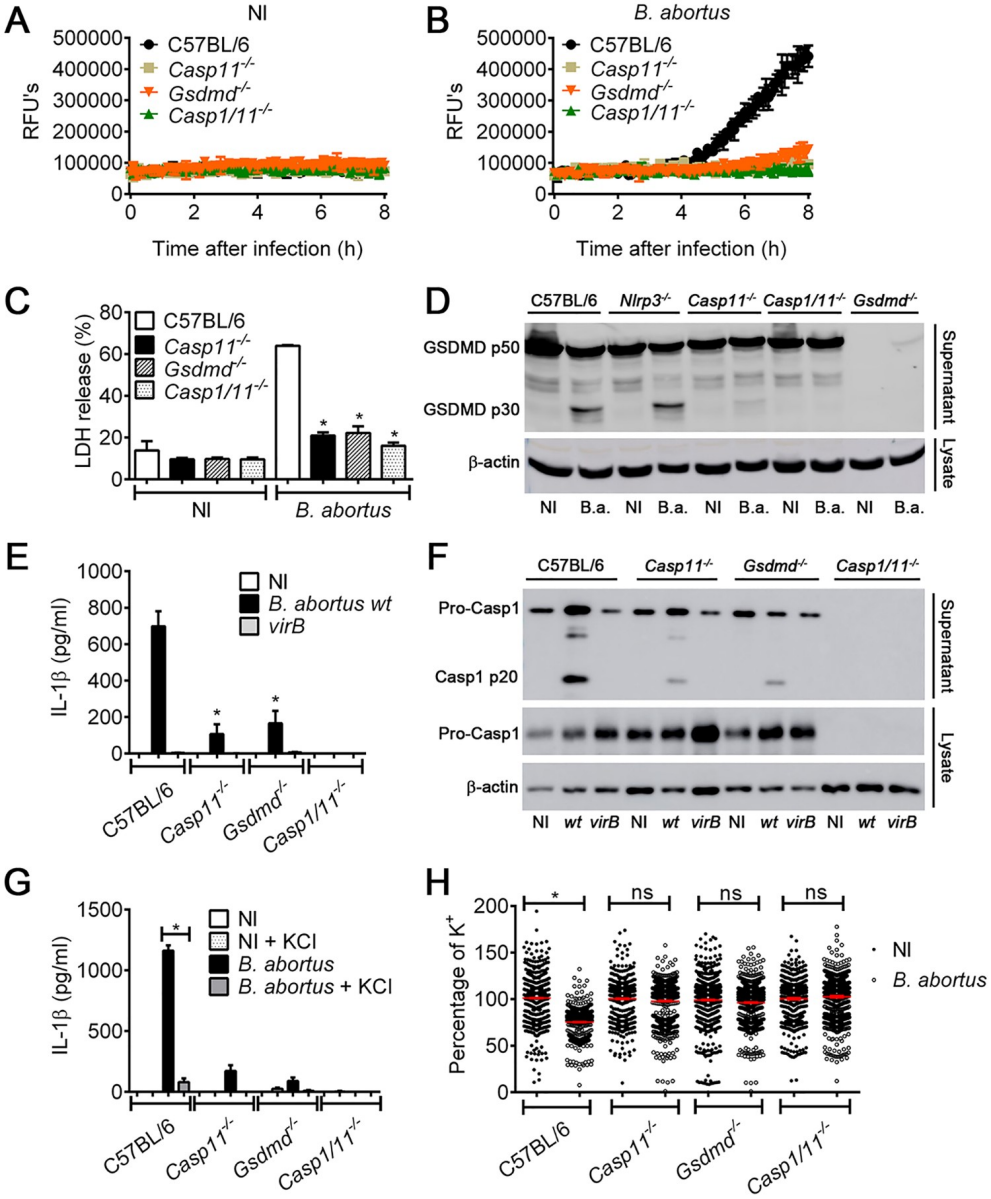
Gasdermin-D (GSDMD) is required to induce pyroptosis and NLRP3 inflammasome activation in response to *B*. *abortus*. BMDMs obtained from C57BL/6, *Casp11*^-/-^, *Gsdmd*^*-/-*^ and *Casp1/11*^*-/-*^ were left uninfected (NI) or stimulated with *B*. *abortus*. BMDMs primed with *E*. *coli* LPS (1 μg/ml) for 4h were left uninfected (A) or infected with *B*. *abortus* (B) at an MOI of 100. Pore formation was assessed fluorimetrically in real time by the uptake of propidium iodide (relative fluorescence units) into the nucleus of the permeabilized BMDMs. (C) Primed BMDMs were left uninfected (NI) or infected with *B*. *abortus* at an MOI of 100 for 8 hrs prior to the assessment of extracellular LDH. Values represent the percentage of LDH released compared with cells lysed with Triton X-100. *p < 0.05 compared with C57BL/6 cultures infected with *B*. *abortus*. (D) Immunoblot showing GSDMD p50 and p30 in lysates of primed BMDMs obtained from C57BL/6, *Nlrp3*^-/-^, *Casp11*^-/-^, *Casp1/11*^*-/-*^ and *Gsdmd*^*-/-*^ infected with *B*. *abortus* at an MOI of 100 for 8 hrs. (E) BMDMs were left uninfected (NI) or infected with *B*. *abortus* or mutant *virB2* at an MOI of 100 for 17 hrs of infection. Supernatants were submitted to ELISA to estimate the concentration of IL-1β. (F) The same culture supernatants and lysates harvested 17 hrs postinfection were separated by SDS-PAGE, blotted and probed with a monoclonal anti-caspase-1 p20 subunit Ab. Equal loading was controlled by measuring β-actin in the corresponding cell lysates. (G) BMDMs were incubated in a medium containing 80 mM KCl 1 h before infection. Then, BMDMs were infected with *B*. *abortus* at an MOI of 100 in the same medium for 17 hrs. IL-1β in the supernatant were measured with mouse IL-1β ELISA kits. Statistically significant difference between BMDMs treated with KCl versus untreated was denoted by an asterisk for p<0.05. (H) Assessment of intracellular K+ levels, as measured by the fluorescent K+ probe APG-2. BMDMs were uninfected (NI) or infected for 6 hrs with *B*. *abortus* at an MOI of 100. Each dot represents the percentage of APG-2 fluorescence intensity in relation to the average fluorescence of control cells, and the red bars represent the mean of all analyzed cells. Statistically significant difference between infected BMDMs versus NI was denoted by * p<0.05. The graphs are representative of two independent experiments. NI: uninfected; *B*.*a*.: *B*. *abortus*; RFU: relative fluorescence unit; *wt*: *B*. *abortus* wild-type; *virB2*: mutant *B*. *abortus*. ns:statistically not significant.

Recently, the identification of a protein termed Gasdermin-D (GSDMD) contributed to the elucidation of the mechanism of pore formation involved in pyroptosis [23, 24, 28, 54–56]. Gasdermin-D acts as a substrate of caspase-11, and once it is cleaved, the N-terminal fragment is recruited to the cell membrane forming pores. Thus, as we observed that *B*. *abortus* is able to trigger pyroptosis in macrophages, we assessed the requirement of GSDMD for pore formation in response to *B*. *abortus*. First, BMDMs obtained from C57BL/6, *Casp11*^*-/-*^, *Gsdmd*^*-/-*^ and *Casp1/11*^*-/-*^ mice were left uninfected or infected with *B*. *abortus*. By evaluating propidium iodide uptake in real time during 8 h of infection, we found that BMDMs from C57BL/6 mice formed pores whereas BMDMs from *Gsdmd*^*-/-*^, *Casp11*^*-/-*^ and *Casp1/11*^*-/-*^ mice failed to form pores in response to *B*. *abortus* (Fig 7B). As expected, non-infected cells were unable to form pores (Fig 7A). It suggests that GSDMD is important to pore formation in response to *B*. *abortus*. Once the GSDMD pore is formed, osmotic pressure leads to water influx inducing cell swelling and consequent membrane disruption, releasing cytosolic content as LDH. Thus, to further evaluate GSDMD role during pyroptosis induced by *B*. *abortus*, we quantified the release of LDH in cell culture supernatants. BMDMs obtained from C57BL/6, *Casp11*^*-/-*^, *Gsdmd*^*-/-*^ and *Casp1/11*^*-/-*^ mice were infected with *B*. *abortus* and after 8 h LDH was measured in supernatants. We found that *B*. *abortus* triggered higher percentage of LDH release in C57BL/6 BMDMs compared to *Gsdmd*^*-/-*^, *Casp11*^*-/-*^ and *Casp1/11*^*-/-*^ cells (Fig 7C). These data support the pore formation assay results, suggesting that GSDMD and caspase-11 are essential to pyroptosis in response to *B*. *abortus*.

Active caspase-11 cleaves GSDMD to separate the regulatory p20 subunit from the cytotoxic p30 subunit, which oligomerizes into the lipid cell membrane forming a pore that culminates in a pyroptosis event. As we observed that *B*. *abortus* triggers pyroptosis dependent of GSDMD, we asked whether caspase-11 was able to cleave GSDMD in response to *B*. *abortus* infection. We infected BMDMs from C57BL/6, *Casp11*^*-/-*^, *Gsdmd*^*-/-*^, *Nlrp3*^*-/-*^ and *Casp1/11*^*-/-*^ mice with *B*. *abortus* and after 17 h, supernatant was harvested and submitted to western blotting. We found that C57BL/6 and *Nlrp3*^*-/-*^ BMDMs were fully able to cleave GSDMD in its active p30 subunit (Fig 7D). However, in *Casp11*^*-/-*^ and *Casp1/11*^*-/-*^ cells GSDMD cleavage was abrogated. As expected, *Gsdmd*^*-/-*^ BMDMs did not express GSDMD protein. Thus, this result indicates that caspase-11 is pivotal to GSDMD cleavage in response to *B*. *abortus*. In addition, NLRP3 was dispensable to GSDMD cleavage. Next, we investigated the role of GSDMD in IL-1β secretion and caspase-1 activation. We infected BMDMs from C57BL/6, *Gsdmd*^*-/-*^, *Casp11*^*-/-*^ and *Casp1/11*^*-/-*^ mice with *B*. *abortus* for 17 hours. The secretion of IL-1β and caspase-1 activation was evaluated in the supernatant of these cells. We observed that *Gsdmd*-deficient BMDMs further resembled *Casp11*-deficient cells presenting reduced levels of IL-1β secretion and the active form of caspase-1 (p20) in comparison to C57BL/6 macrophages (Fig 7E and 7F). As expected, *Casp1/11*^*-/-*^ BMDMs did not secrete IL-1β or express caspase-1. As indicated in the literature, GSDMD pores allow the efflux of ions such as potassium as well as limited secretion of small cytosolic proteins that fit through these pores, such as IL-1β [27]. Despite the wide variety of stimuli that trigger NLRP3 (e.g., reactive oxygen species, release of oxidized mitochondrial DNA, lysosomal cathepsins and bacterial RNA) potassium efflux has emerged as a point of convergence essential to NLRP3 inflammasome activation [30–35]. Thus, we decided to investigate the requirement of potassium efflux for NLRP3 activation in response to *B*. *abortus*. We submitted BMDMs from C57BL/6, *Gsdmd*^*-/-*^, *Casp11*^*-/-*^ and *Casp1/11*^*-/-*^ mice to a medium containing high K^+^ concentration and after 1 h, cells were infected with *B*. *abortus*. After 17 h, secretion of IL-1β in the supernatant was evaluated. We observed a significant reduction in the secretion of IL-1β in C57BL/6, *Casp11*^*-/-*^ and *Gsdmd*^*-/-*^ BMDMs when cells were incubated in high-K^+^ media (Fig 7G). As expected, IL-1β was not processed in BMDMs from *Casp1/11*^*-/-*^ mice. Increased extracellular [K^+^] prevented NLRP3 activation, suggesting a great importance of potassium efflux to inflammasome activation in response to *B*. *abortus*. Next, we tested whether GSDMD and caspase-11 were required for potassium efflux in response to *B*. *abortus*. We found that intracellular potassium concentration decreased inside C57BL/6 BMDMs in response to *B*. *abortus* infection whereas in *Casp11*^*-/-*^, *Gsdmd*^*-/-*^ and *Casp1/11*^*-/-*^ macrophages it remained at similar levels as observed in the non-infected controls (Fig 7H).

In summary, these data suggest that pyroptosis which is dependent on caspase-11 and GSDMD are central to potassium efflux and, consequently, to NLRP3 inflammasome activation in response to *B*. *abortus*.

### GSDMD and caspase-11 are essential to control *B*. *abortus* infection and regulates neutrophil, macrophage and dendritic cell recruitment

**Fig 8:**
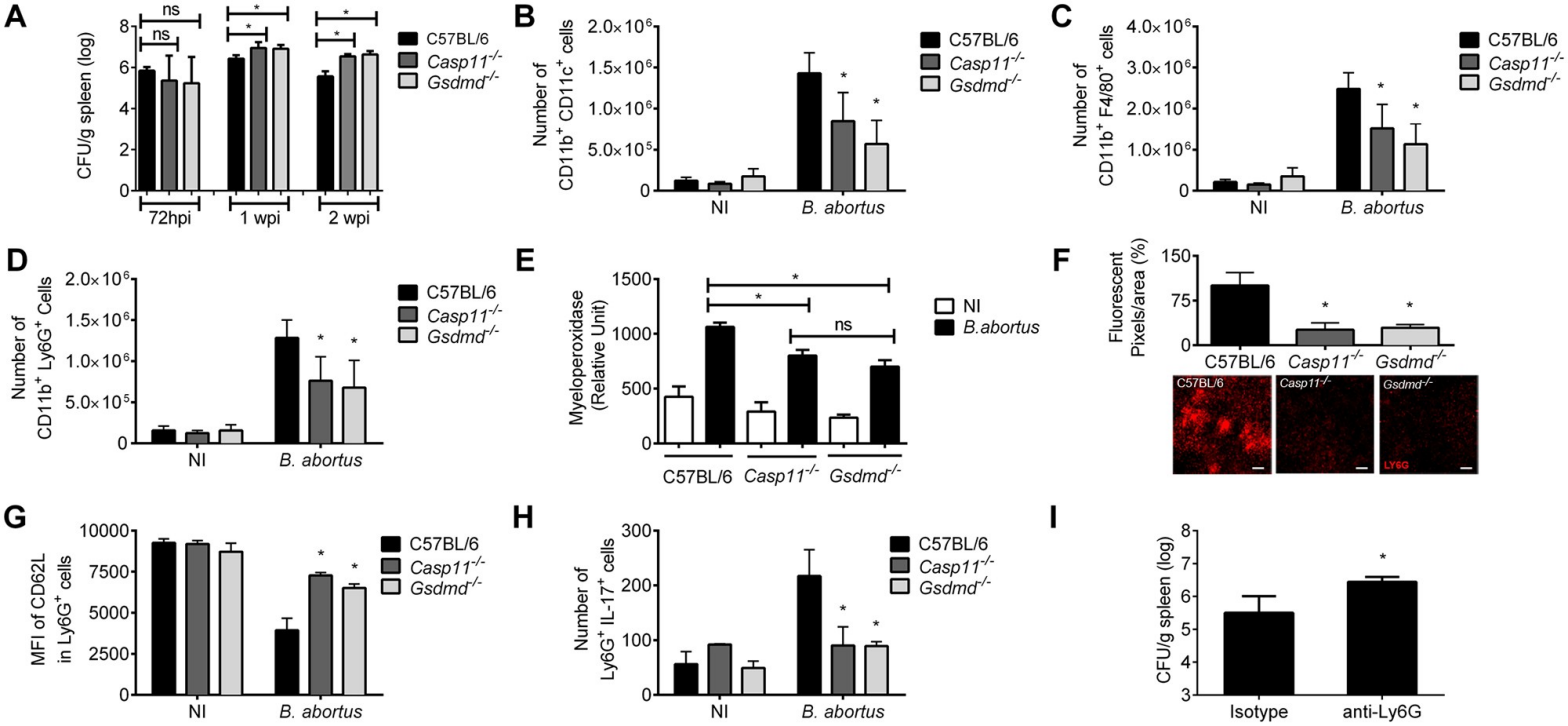
GSDMD is important to control *B*. *abortus* infection and regulates innate immune cell recruitment. (A) C57BL/6, *Gsdmd*^*-/-*^ and *Casp11*^*-/-*^ mice were infected intraperitoneally with 1 x 10^6^ CFU of *B*. *abortus*. Mice were sacrificed at 72h, 1 and 2 weeks postinfection, and diluted spleen homogenates were added to BB medium agar plates for CFU determination. (B-D) Spleen cells from infected C57BL/6, *Gsdmd*^*-/-*^ and *Casp11*^*-/-*^ mice were stained ex-vivo for flow cytometry analysis. Cells were assessed for CD11b^+^ CD11c^+^ (B), CD11b^+^ F4/80^+^ (C) and CD11b^+^ Ly6G^+^ (D). Data are mean ± SD of five mice/group. (E) Splenic homogenates from mice C57BL/6, *Casp11*^*-/-*^ and *Gsdmd*^*-/-*^ infected with *B*. *abortus* were submitted to a myeloperoxidase (MPO) activity assay. Data are mean ± SD of five mice/group. (F) C57BL/6, *Casp11*^*-/-*^ and *Gsdmd*^*-/-*^ mice were infected with *B*. *abortus*, and 3 days post-infection were inoculated i.v. with Ly-6G PE antibody. Representative images show whole organ *ex-vivo* confocal images from spleens from each group. Percentage of red fluorescent pixels per organ area is also shown. Scale bar = 100 μm. (G) Analysis of CD62L MFI (median of fluorescence intensity) in C57BL/6, *Casp11*^*-/-*^ and *Gsdmd*^*-/-*^ Ly6G^+^ cell population, when stimulated with *B*. *abortus* or medium alone (NI). (H) Number of Ly6G^+^ cells expressing IL-17 in 1x10^6^ splenocytes of C57BL/6, *Casp11*^*-/-*^ and *Gsdmd*^*-/-*^ mice infected two weeks with *B*. *abortus* (MOI:100). (I) Analysis of *Brucella* CFU in neutrophils depleted mice. Prior to and during infection, mice were treated with isotype control or with anti-Ly6G antibody. Spleens were excised at day 7 postinfection and bacterial load was measured. * *p*<0.05, compared to wild-type mice. The graphs are representative of two independent experiments. DCs: dendritic cells; ns: statistically not significant; NI: non-infected.

Since GSDMD triggered pyroptosis was associated to bacterial clearance [36], we investigated the role of GSDMD in controlling *B*. *abortus* infection. We infected C57BL/6, *Gsdmd*^*-/-*^ and *Casp11*^*-/-*^ mice intraperitoneally with *B*. *abortus* and after 72h, 1 and 2 weeks, bacterial CFU in spleens were evaluated. Bacterial load recovery was higher in *Gsdmd*^*-/-*^ and *Casp11*^*-/-*^ mice in comparison to C57BL/6 at 1 and 2 weeks postinfection (Fig 8A). However, no difference in bacterial counts was observed at 72h following *Brucella* infection. Further, we measured *Brucella* intracellular replication in C57BL/6, *Gsdmd*^*-/-*^ and *Casp11*^*-/-*^ macrophages at 2, 24 and 48 hrs *in vitro*. No difference in intracellular CFU was detected among macrophages from tested mouse groups (S3 Fig). This finding suggests that lack of caspase-11 and GSDMD does not affect *Brucella* entry in macrophages at the initial colonization stage. Additionally, we infected C57BL/6, *Casp11*^*-/-*^, *Casp1*^*-/-*^*Casp11*^*Tg*^, *Casp1/11*^*-/-*^ and *Nlrp3*^*-/-*^ mice intraperitoneally with *B*. *abortus*. After 2 weeks of infection, bacterial CFU were determined from spleen homogenate. *Casp1*^*-/-*^*Casp11*^*Tg*^ and *Nlrp3*^*-/-*^ were as resistant as C57BL/6 mice (S4 Fig). In contrast, bacterial loads were approximately 1 log higher in *Casp11*^*-/-*^ and *Casp1/11*^*-/-*^ mice compared with C57BL/6 animals. These results demonstrate that GSDMD and caspase-11 deficiency but not caspase-1 are important to *B*. *abortus* control in mice.

As we observed that *Gsdmd*^*-/-*^ mice are more susceptible and that GSDMD is involved in pyroptosis in response to *B*. *abortus*, we decided to investigate the mechanism involved in the susceptibility of GSDMD mice. To further evaluate that, we assessed whether GSDMD- and caspase-11- deficient mice have a deficiency in the recruitment of immune cell populations. C57BL/6, *Casp11*^*-/-*^ and *Gsdmd*^*-/-*^ mice were infected with *B*. *abortus* and after 2 weeks we analyzed the numbers of neutrophils, macrophages and dendritic cells by flow cytometry. We observed higher numbers of neutrophils, dendritic cells and macrophages in the spleen of C57BL/6 mice infected with *B*. *abortus* compared to non-infected mice (Fig 8B–8D). However, when we analyzed these cells populations in the spleens of *Gsdmd*^*-/-*^ and *Casp11*^*-/-*^ mice infected with *B*. *abortus*, we observed a reduction in numbers of neutrophils, dendritic cells and macrophages compared to C57BL/6 mice. Additionally, we submitted splenic homogenates from C57BL/6, *Casp11*^*-/-*^ and *Gsdmd*^*-/-*^ mice infected with *B*. *abortus* to a myeloperoxidase (MPO) activity assay and measurement of KC levels to corroborate whether *Gsdmd*- and *Casp11*- deficient mice showed less neutrophil recruitment. We observed MPO reduced activity (Fig 8E) and diminished KC levels (S5 Fig) in *Gsdmd*^*-/-*^ and *Casp11*^*-/-*^ splenic homogenates from mice infected with *B*. *abortus* compared to homogenates from C57BL/6 mice. To confirm this deficiency in neutrophil recruitment, we performed confocal microscopy analysis *ex vivo* of mouse spleens 72 h after *B*. *abortus* infection. Clearly, we observed a reduced influx of neutrophils in *Gsdmd*^*-/-*^ and *Casp11*^*-/-*^ spleens labeled with anti-Ly6G antibody when compared to wild-type animals (Fig 8F). These findings support the hypothesis that GSDMD and caspase-11 play a role in neutrophil recruitment in response to *B*. *abortus*. To determine whether these neutrophils are activated, we measured CD62L surface expression levels in *Gsdmd*^*-/-*^ and *Casp11*^*-/-*^ mice by flow cytometry, a L-selectin marker of neutrophil activation. The levels of CD62L on Ly6G+ cells were higher in *Gsdmd*^*-/-*^ and *Casp11*^*-/-*^ infected animals compared to C57BL/6, what is related to less activated neutrophils (Fig 8G). Down-regulation of CD62L surface expression in neutrophils is characteristic of cell activation [57]. Additionally, we measured the number of IL-17 expressing Ly6G+ cells in mouse spleens. Two-weeks post-infection, *Gsdmd*^*-/-*^ and *Casp11*^*-/-*^ animals showed reduced production of IL-17 within the Ly6G+ cell population compared to C57BL/6 animals (Fig 8H). To determine the role of neutrophils in the control of *Brucella* infection, we treated mice with anti-Ly6G antibody for one week. Depletion of neutrophils in wild-type animals infected with *Brucella* renders mice more susceptible to bacterial replication *in vivo* (Fig 8I and S6 Fig). Taken together, these data suggest that caspase-11 and GSDMD play a role in *B*. *abortus* infection restriction in mice and mediate neutrophil, macrophage and dendritic cell recruitment and activation.

## Discussion

Lipopolysaccharides (LPS) of Gram-negative bacteria are the major component of its outer membrane and crucial to the recognition of bacteria by immune cells [58]. They are recognized by TLR4, drive the induction of proinflammatory cytokines such as tumor necrosis factor (TNF-α) [59] and are great inductors of septic shock [58]. However, pathogenic bacteria developed strategies to escape the recognition by the immune system to establish an infection inside the host. One of these strategies is the modification of its LPS to avoid effective recognition by TLR4 [60]. *B*. *abortus* is an example among Gram-negative bacteria that contains a low immunostimulatory LPS with long-chain fatty acid, being an important virulence factor [61–63]. In that context, caspase-11 arises as a second barrier for LPS recognition acting as an intracellular receptor to promote cytoplasmic surveillance [15–17]. Once activated, it leads to pyroptosis and NLRP3 inflammasome activation and consequent caspase-1 activation and proinflammatory cytokines release, being critical to innate immunity against Gram-negative bacteria [13, 15, 16, 18]. Therefore, in this study, we investigated whether *B*. *abortus* were able to activate this non-canonical caspase-11 inflammasome. Here, we demonstrated that caspase-11 is important to caspase-1 activation and IL-1β and IL-1α secretion in response to *B*. *abortus*. We also evaluate if *B*. *abortus* LPS was the PAMP responsible for activation of the non-canonical inflammasome. Surprisingly, we observed that purified *B*. *abortus* LPS was sufficient to drive caspase-11 non-canonical inflammasome activation. Although *B*. *abortus* LPS escapes cell surface surveillance by TLR4, it cannot escape caspase-11 cytoplasmic control. This is distinct from other bacteria, such as *Francisella novicida*, that modify their LPS, and escape immunosurveillance by both TLR4 and caspase-11 [15]. Hence, the caspase-11 pathway seems to be important to control *B*. *abortus* infection. The recognition of LPS by caspase-11 occurs when this molecule is hexa-acylated [16]. *B*. *abortus* LPS contains long-chain fatty acid, nevertheless it is hexa-acylated [64]. Moreover, it is already reported that *Legionella pneumophila*, whose LPS is hexa-acylated with long-chain fatty acid, activates the non-canonical inflammasome [65], likewise we observed here for *B*. *abortus*. Even though we used in this study *E*. *coli* LPS primed-macrophages to show caspase-11 activation and pyroptosis induced by *B*. *abortus*, unprimed cells also showed similar phenotype. However, once these cells are primed prior to the moment of infection, inflammasome proteins are already highly expressed, cells are synchronized and ready to respond to a second signal, thus inducing higher levels of pore formation and caspase-11 activation compared to unprimed cells. Regarding macrophages transfected with *Brucella* LPS, PAM3CSK priming was required to activate the caspase-11/pyroptosis pathway. This fact makes sense, since during infection other *Brucella* PAMPs such as lipoproteins may deliver the first signal to activate the cell and when bacterial LPS is release into the cytoplasm caspase-11 is ready to recognize it.

Pilla et al. suggested that mouse chromosome 3 GBPs possibly act in collaboration with caspase-11 in the recognition of bacterial LPS with structural differences in the lipid A moiety of *L*. *pneumophila* [21]. Furthermore, Santos et al., confirmed that GBP^chr3^ proteins facilitate the interaction of LPS with caspase-11 [20]. In addition, previous studies including one from our group demonstrated that GBPs can associate with pathogen-containing vacuoles contributing to its lysis and resulting in the release of bacterial PAMPs in the cytoplasm [22, 66]. Here we observed that GBP^chr3^ proteins are required for caspase-11 activation and pyroptosis upon macrophage infection with whole *B*. *abortus* or transfected with its purified LPS. Accordingly, our data support the idea that GBPs contribute to BCV lysis, as previously shown by our group, but also these molecules can contribute to the recognition of bacterial LPS by caspase-11. Additionally, Santos et al. showed that the role of Gbp^chr3^ proteins mediating interaction of LPS with caspase-11 are especially observed when LPS is incorporated within liposomal membranes [20]. Indeed, here we used FuGENEHD reagent in the transfections which incorporates LPS in liposomal vesicle which mimics the LPS-containing membranes. Additionally, to determine which GBP from the mouse chromosome 3 would be involved in caspase-11 sensing of *Brucella* LPS, we knocked down *GBP1*, *GBP3*, *GBP5*, *GBP7* by siRNA in C57BL/6 macrophages and used GBP2 KO cells. Lack of *GBP5* expression but not other GBPs resulted in reduced IL-1β secretion and LDH release in macrophages transfected with *Brucella* LPS. These findings suggest that GBP5 is the molecule responsible for the phenotype observed in GBP^chr3^ KO mice related to caspase-11 recognition of *Brucella* LPS. More recently, our research group identified that miR-21a-5p led to downregulation of *GBP5* expression in macrophages infected with *Brucella* and increased bacterial counts in macrophages [67]. This study highlights the importance of GBP5 regulation by a miRNA in macrophage susceptibility to *Brucella* infection.

In the last few years, great progress in comprehension of the pyroptosis mechanism was achieved. Studies demonstrated that once caspase-11 is activated, it cleaves GSDMD into two domains: a C-terminal p20 domain and an N-terminal p30 domain which oligomerizes and inserts into the membrane forming a pore [23, 24, 27, 28]. Since water can enter into cells through these pores, an osmotic imbalance is created leading to cell death [27]. In the case of *Brucella*, previous reports established that smooth virulent *Brucella* inhibit macrophage cell death whereas rough attenuated strain induces apoptosis via caspase-2 activation [68–71]. In contrast, another study observed that smooth *B*. *melitensis* induced apoptosis in Raw264.7 macrophage cell lines via ROS production [72]. Additionally, several reports have observed that *B*. *abortus* smooth strain 2308 induced apoptotic cell death in dendritic cells, astrocytes and T lymphocytes [73–75]. In our study, we observed pore formation and confirmed cell death using LDH release assay suggesting that *B*. *abortus* triggers caspase-11/GSDMD-dependent pyroptosis. Here, we infected BMDMs using opsonized *B*. *abortus* in order to increase phagocytosis and synchronize the infection, a different protocol used by other *Brucella* investigators. Notably, this strategy is the one which better mimics the *in vivo* infection and has been extensively used in other studies involving other pathogens and pyroptosis [76–80]. Hence, it may explain these discrepancies observed in our study in comparison to previous reports. More recently, Lacey et al. studying the role of inflammasomes in *Brucella*-induced arthritis concluded that the smooth *Brucella* strain induces pyroptosis in macrophages via caspase-1/caspase-11 pathway, confirming our results [81]. Furthermore, the pyroptosis event also seems to be strongly related to restricting infection *in vivo*. We observed that mice deficient in caspase-11 and GSDMD that are involved in pyroptosis are more susceptible to *Brucella* infection compared to wild-type animals, suggesting that *B*. *abortus* triggers pyroptosis and this event is important to control infection. Recently others reported that pyroptosis leads to secretion of molecules such as IL-1β, IL-1α and eicosanoids which recruit neutrophils to the site of infection promoting phagocytosis of infected cells and contributing to restricting infection [29, 36]. Indeed, here we observed lower neutrophil, macrophage and dendritic cell recruitment in the spleen of *Casp11*^*-/-*^ and *Gsdmd*^*-/-*^ mice infected with *B*. *abortus*. We hypothesize that this cell recruitment and activation deficiency could be one of the mechanisms to explain the increased bacterial burden observed in *Casp11*^*-/-*^ and *Gsdmd*^*-/-*^ mice in response to this bacterium. To confirm that, we depleted neutrophils from infected wild-type animals and our results demonstrated that neutrophil depletion enhanced mouse susceptibility to *Brucella* infection. Therefore, we speculate that caspase-11/GSDMD-dependent pyroptosis contributes to immune cell recruitment and activation in response to *B*. *abortus* and this process may promote infection control in mice, although formal validation is still required. Additionally, in a previous study from our group we have shown that lack of IL-1R renders mice more susceptible to *Brucella* infection [52]. So, reduced production of IL-1β in *Casp11*^*-/-*^ and *Gsdmd*^*-/-*^ mice is another possible mechanism to enhance susceptibility to infection. IL-1α release has also been related to neutrophil recruitment and infection control in response to other bacteria such as *L*. *pneumophila* [82]. However, although we observed here that *Casp11*^*-/-*^ BMDMs released reduced IL-1α levels, IL-1α-deficient mice did not show increased bacterial load after 2 weeks of infection when compared to wild-type animals in response to *B*. *abortus* (S7 Fig). Thus, IL-1α does not seem to be linked to infection control in response to *B*. *abortus*. In summary, caspase-11 and GSDMD KO susceptibility to *Brucella* is triggered by a multifaceted inflammatory response against this bacterial infection.

**Fig 9:**
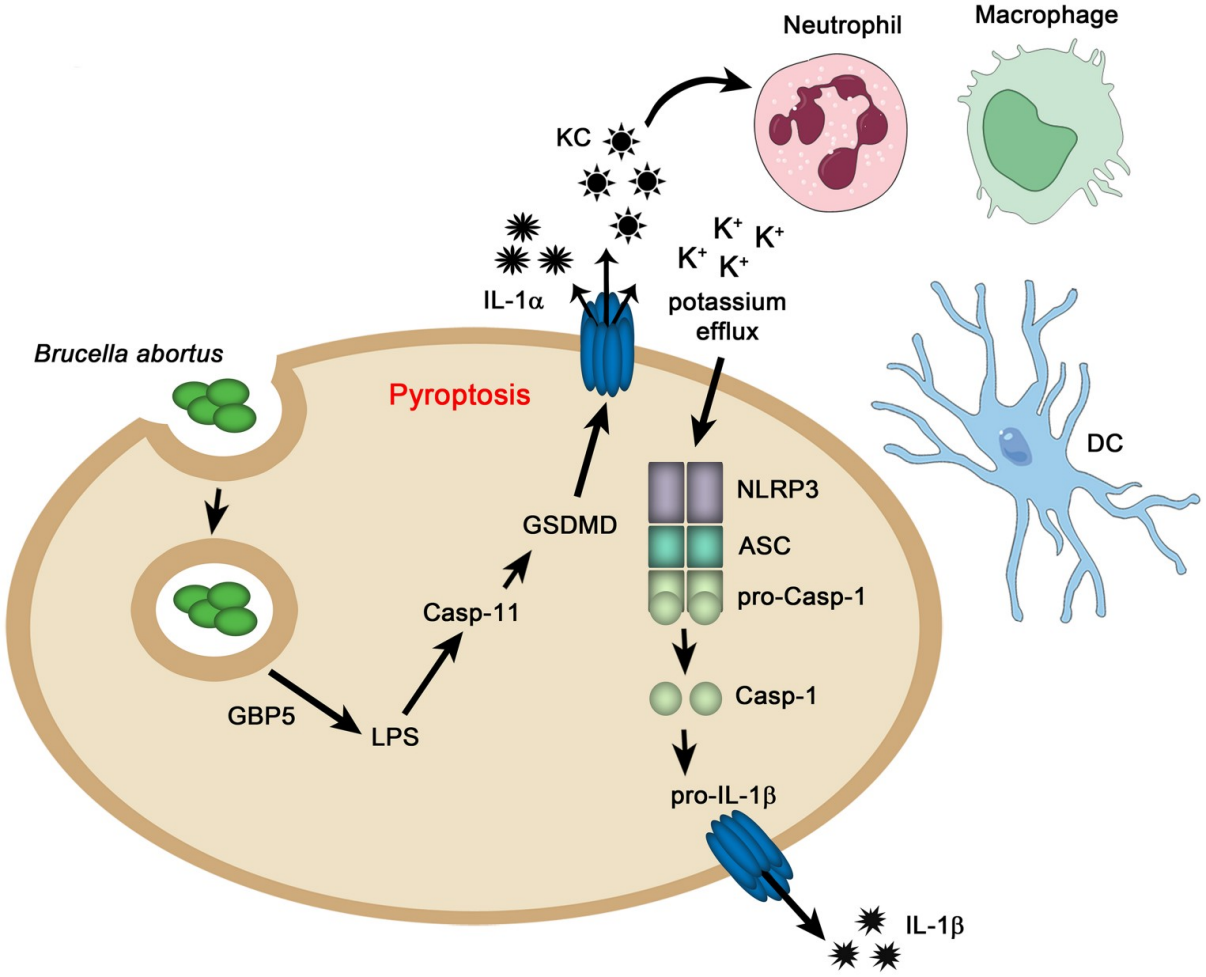
Schematic model proposed for non-canonical inflammasome activation in response to *B*. *abortus* infection. Phagocyted *B*. *abortus* secretes effector proteins in the macrophages cytosol and stablishes its BCV (*Brucella* containing vacuole). BCV lysis and recognition of *B*. *abortus* LPS by caspase-11 occur with the participation of GBP5 protein. Activated caspase-11 cleaves GSDMD in its p20 and p30 forms. Once cleaved, GSDMD p30 subunit triggers pyroptosis that promotes K+ efflux contributing to NLRP3 inflammasome activation. This inflammasome activation culminates in caspase-1 activation and IL-1β secretion. Furthermore, pyroptosis induced by GSDMD releases other cytokines such as IL-1α and KC that contributes to recruitment of immune cells as neutrophils, dendritic cells and macrophages that may participate on the restriction of *B*. *abortus* infection in mice.

Overall, our results lead to a model in which *B*. *abortus* is phagocytized by macrophages and establishes its BCV (*Brucella* containing vacuole) to replicate. GBP^chr3^ proteins, mainly GBP5, contributes to BCV lysis and recognition of *B*. *abortus* LPS by caspase-11 leading to cell activation. Once activated, caspase-11 cleaves GSDMD into its p20 and p30 forms. Cleaved p30 GSDMD subunit drives pyroptosis promoting K^+^ efflux which contributes to NLRP3 inflammasome activation leading caspase-1 activation and IL-1β secretion. Furthermore, the pyroptosis event possibly contributes to proinflammatory molecule secretion that drives neutrophil, dendritic cell and macrophage recruitment and activation, which participate to restrict *B*. *abortus* infection in mice (Fig 9).

The results of this study provide relevant information to the elucidation of a pathway of bacterial sensing involved in the recognition of *B*. *abortus* LPS and potential mechanisms of host protection against this stealthy pathogen. Furthermore, these findings advance in the comprehension of bacterial pathogenesis and contribute to the future development of drugs or vaccines to control brucellosis.

## Material and methods

### Bacterial strains

*Brucella abortus* strain 2308 was obtained from our laboratory collection. The Δ*virB2 B*. *abortus* mutant strain used in this study was obtained by allelic exchange of the *virB2* gene, generating a polar deletion of *virB2* and it was kindly provided by Dr. Renato de Lima Santos from the Federal University of Minas Gerais (UFMG), Brazil [83]. All bacteria were grown in Brucella broth medium (BD Pharmingen, San Diego, CA) for 1 d at 37°C under constant agitation. The culture OD at 600 nm was measured in a spectrophotometer to determine the bacterial number in the solution.

### Ethics statement

This study was carried out in strict accordance with the Brazilian laws 6638 and 9605 in Animal Experimentation. The protocol was approved by the Committee on the Ethics of Animal Experiments of the Federal University of Minas Gerais (Permit Number: CETEA #128/2014).

### Mice

Wild-type C57BL/6 mice were purchased from the Federal University of Minas Gerais (UFMG). *Nlrp3*^*-/-*^ and *Casp1/11*^*-/-*^ were described previously and backcrossed to C57BL/6 mice for at least eight generations [3, 84]. *Casp11*^*-/-*^, *Gsdmd*^*-/-*^, *Gbp2*^*-/-*^ and *Gbp*^*chr3-/-*^ mice were generated in the C57BL/6 background [13, 23, 85, 86]. *Casp1*^*−/−*^*Casp-11*^*Tg*^ mice are *Casp1/11*^*−/−*^ mice expressing a transgene encoding a functional copy of the caspase-11 allele as previously described [13]. The animals were maintained at UFMG and used at 6–9 wk of age.

### Generation of bone marrow-derived macrophages (BMDMs)

Macrophages were derived from bone marrow of indicated mice in L929 cell–conditioned medium as previously described [80]. Briefly, bone marrow cells were harvested from femurs and differentiated with DMEM (Life Technologies, Carlsbad, CA) containing 20% fetal bovine serum (Life Technologies, Carlsbad, CA) and 30% L-929 cell-conditioned medium (LCCM), 15 mM Hepes (Life Technologies, Carlsbad, CA) and 100 U/ml penicillin-streptomycin (Life Technologies, Carlsbad, CA) at 37°C with 5% CO2 [80]. BMDMs were seeded at 5 x 10^5^ cells/well in 24-well plates and cultivated in DMEM supplemented with 1% FBS and 15 mM Hepes.

### *B*. *abortus* LPS transfection in BMDMs

BMDMs were seeded into 24-well plates (5 × 10^5^ cells/well). The cells were primed with PAM3CSK (1 μg/ml) for 6 h. Two solutions were made to perform the *B*. *abortus* LPS transfection one containing DMEM medium without FBS and with FuGENEHD (Promega, Madison, USA); and other containing DMEM medium without FBS and with *B*. *abortus* LPS (kindly provided by Dr. Ignacio Moriyón at Universidad de Navarra, Pamplona, Spain). These solutions were mixed and kept for 15 min at room temperature before the addition to the cells. After 17 h of transfection, supernatant and lysates were collected to be submitted to Western blotting and ELISA.

### Propidium iodide uptake assay and Ab generation

Pore formation in BMDMs was determined by quantifying propidium iodide uptake as previously described [76]. BMDMs were seeded into black 96-well plates (1 × 10^5^ cells/well) and pre-stimulated with *E*. *coli* LPS (1 μg/ml) or PAM3CSK (500 ng/ml) during 4 or 6 h, respectively. The cells were submitted to RPMI 1640 media lacking phenol red with 15 mM HEPES and 0.38 g/l NaHCO_3_ supplemented with 10% (v/v) FBS and 6 μg/ml propidium iodide. BMDMs were immediately infected or transfected. Infections were performed with *Brucella abortus* wild-type at an MOI of 100 for 8 h. Transfections with *B*. *abortus* LPS were performed using FuGENEHD (Promega, Madison, USA) as described above and propidium iodide uptake was measured at 24 h. Throughout infection/transfection, the plates were incubated at 37°C in a FlexStation 3 microplate reader (Molecular Devices, Sunnyvale, CA), and propidium iodide fluorescence was measured every 1 h. During the infections, bacteria were opsonized with a mouse polyclonal Ab (anti-*B*. *abortus*, 1:1000 dilution) in order to ensure greater efficiency of bacterial phagocytosis. This polyclonal Ab was generated by injecting 1x10^6^ heat-killed bacteria/mouse. Animals were injected three times during a 15-d interval; then, the serum of each mouse was tested for the presence of the specific Ab and stored at −80°C.

### Lactate dehydrogenase release assay

BMDMs were seeded into 24-well plates (5 × 10^5^ cells/well) and infected with *B*. *abortus* at an MOI of 100. Infections were performed in DMEM media lacking phenol red. After 8 h of infection, supernatants were harvested for analysis of lactate dehydrogenase (LDH) release by dying cells. Total LDH was determined by lysing the cultures with Triton X-100. LDH was quantified using the CytoTox 96 LDH-release kit (Promega, Madison, WI), according to the manufacturer’s instructions.

### Active caspase-11 pull-down assay

BMDMs were seeded into 6-well plates (1 × 10^7^ cells/well). The media of BMDMs were replenished with fresh media containing 20 μM biotin-VAD-FMK (Enzo), a pan-caspase inhibitor, 15 min before infection. BMDMs were infected with *B*. *abortus* at an MOI of 100 or transfected with *B*. *abortus* LPS as described above. Infected/transfected BMDMs were lysed in RIPA buffer (10 mM Tris-HCl (pH 7.4), 1 mM EDTA, 150 mM NaCl, 1% Nonidet P-40, 1% (w/v) sodium deoxycholate and 0.1% (w/v) SDS) supplemented with a protease inhibitor cocktail (Thermo-Fisher). Cleared lysates were incubated overnight with streptavidin–agarose beads (Novex) and thoroughly rinsed with RIPA buffer. Bound proteins were eluted by re-suspension in Laemmli sample buffer, boiled for 5 min and subjected to SDS-PAGE analysis and Western blotting as described above.

### Real-time PCR

RNA was extracted from BMDMs with TRIzol reagent (Invitrogen, Carlsbad, CA) to isolate total RNA in accordance with the manufacturer’s instructions. Reverse transcription of 2 μg of total RNA was performed using Illustra Ready-To-Go RT-PCR Beads (GE Healthcare, Chicago, IL) according to the manufacturer’s directions. Real-time RT-PCR was performed using 23 SYBR Green PCR master mix (Applied Biosystems, Foster City, CA) on a QuantStudio3 real-time PCR instrument (Applied Biosystems, Foster City, CA). The appropriate primers were used to amplify a specific fragment corresponding to specific gene targets as follows: β-actin, forward, 5’- GGCTGTATTCCCCTCCATCG-3’, reverse, 5’-CCAGTTGGTAACAATGCCATGT-3’; GBP1, forward, 5’-GAGTACTCTCTGGAAATGGCCTCAGAAA-3’, reverse, TAGATGAAGGTGCTGCTGAGGAGGACTG-3; GBP2, forward, 5’-CTGCACTATGTG ACGGAGCTA-3’, reverse, 5’-CGG AATCGTCTACCCCACTC-3’; GBP3, forward, 5’-CTGACAGTAAATCTGGAAGCCAT-3’, reverse, 5’-CCGTCCTGCAAGACGATT CA-3’; GBP5, forward, 5’-CTGAACTCAGATTTTGTG CAGGA-3’, reverse, 5’-CATCGACATAAGTCAGCACCAG-3’; GBP7, forward, 5’-TCCTGTGTGCCTAGTGGAAAA-3’, reverse, 5’-CAAGCGGTTCATCAAGTAGGAT-3’. All data are presented as relative expression units after normalization to the *β-actin* gene, and measurements were conducted in triplicate.

### Knockdown via small interfering RNA

BMDMs were previously primed with PAM3CSK (500 ng/ml) and after 6 hours, they were transfected with siRNA from siGENOME SMARTpools (Dharmacon, Lafayette, CO) with the GenMute siRNA transfection reagent according to the manufacturer’s instructions (SignaGen, Rockville, MD). siGENOME SMARTpool siRNAs specific for mouse GBP1 (M-040198010005, GBP3 (M-063076-01-0005), GBP5 (M-054703-01-0005), and GBP7 (M-061204-01-0005) were used in this study. A control siRNA pool was used (D-001206-14-05). Forty-six hours after siRNA transfection, cells were transfected with *B*. *abortus* LPS (5 μg/ml) as described above. After 17h, supernatant was collected to measure IL-1β by ELISA and LDH release using the CytoTox 96 LDH-release kit (Promega, Madison, WI), according to the manufacturer’s instructions.

### Flow cytometry analysis

Five mice from each group (C57BL/6, *Casp11*^*-/-*^ and *Gsdmd*^*-/-*^) were infected i.p. with 1 × 10^6^ CFU *B*. *abortus* virulent strain S2308 and sacrificed at 2 weeks postinfection. Spleen cells were harvested and washed twice with sterile PBS. After washing, the cells were adjusted to 1x10^6^ cells in RPMI medium supplemented with 10% fetal bovine serum, 150 U penicillin G sodium and 150 μg streptomycin sulfate per well in a 96-well plate. After, the cells were centrifuged at 1500 rpm for 7 min at 4°C and washed with PBS containing 1% bovine serum albumin (PBS/BSA). The cells were incubated with anti-CD16/CD32 (FcBlock) (1:30 diluted in PBS/BSA) for 20 min at 4°C. The cells were then centrifuged and washed in PBS/BSA and incubated for 20 min at 4°C with a mixture of the following antibodies: rat IgG2a anti-murine F4/80 conjugated to biotin (clone BM8; 1:200); rat IgG2b anti-murine CD11b conjugated to APC-Cy7 (clone M1/70; 1:200); hamster IgG1 anti-murine CD11c conjugated to FITC (clone HL3; 1:200); rat IgG2a anti-murine Ly-6G conjugated to PE (clone 1A8; 1:200) and rat IgG2a anti-murine CD62L conjugated to APC (clone MEL-14; 1:400). All antibodies were obtained from BD Bioscience. The cells were centrifuged and washed again with PBS/BSA and incubated with streptavidin conjugated to PerCP Cy5.5 (1:30) for 20 min at 4°C. To measure IL-17, the cells were centrifuged and washed again with PBS/BSA and fixed and permeabilized using BD Cytofix/Cytoperm reagent (BD Bioscience, San Diego, CA, USA) according to the manufacturer’s instructions. The cells were then incubated with rat IgG2a anti-murine IL-17 conjugated to PE (clone eBio 18F10; 1:30; eBioscience) for 30 min at 4°C. Finally, the cells were washed three times, suspended in PBS buffer and evaluated using Attune Acoustic Focusing equipment (Life Technologies, Carlsbad, CA, USA). The results were analyzed using FloWJo software (Tree Star, Ashland, OR, USA).

### Cytokine measurements

For cytokine determination, BMDMs were seeded at a density of 5 × 10^5^ cells/well in 24-well plates. BMDMs were infected with *B*. *abortus* or *virB2* mutant strain at an MOI of 100 or transfected with *B*. *abortus* LPS, as described above, for 17h. As a positive control, cells were primed with 1 μg/ml of *E*. *coli* LPS (Sigma-Aldrich, St. Louis, MO, USA) for 4h and stimulated with 20μM nigericin sodium salt (Sigma-Aldrich) for 30 minutes. Supernatants were harvested and cytokines were measured with mouse IL-1β, IL-1α, TNF-α and IL-12 ELISA kits (R&D systems, Minneapolis, MN) according to the manufacturer’s instructions. For measurement of KC, five mice from each group (C57BL/6, *Casp11*^*-/-*^ and *Gsdmd*^*-/-*^) were infected intraperitoneally with 1 × 10^6^ CFU *B*. *abortus* virulent strain S2308 and sacrificed at 2 weeks postinfection. Fragments with approximately 100 mg from the harvested spleens were homogenated in 1 ml of cytokines extraction solution (Phosphate-Buffered Saline (PBS) containing an anti-proteases cocktail (0.1 mM PMSF, 0.1 mM benzethonium chloride, 10 mM EDTA e 20 KI aprotinin A) and 0,05% Tween-20) using a tissue homogenator (T10 basic ULTRA-TURRAX, IKA, Germany). Next, homogenates were centrifuged at 10000 rpm for 10 min at 4°C. The supernatants were immediately collected and kept at 80° C to posterior cytokine measurement. KC was measured using ELISA kit (R&D systems, Minneapolis, MN) according to the manufacturer’s instructions.

### Western blot analysis

BMDMs were seeded at a density of 5 × 10^5^ cells/well in 24-well plates. BMDMs were infected with *B*. *abortus* or *virB2* mutant strain at an MOI of 100 or transfected with *B*. *abortus* LPS, as described above, for 17h. As a positive control, cells were primed with 1 μg/ml of *E*. *coli* LPS (Sigma-Aldrich, St. Louis, MO, USA) for 4h and stimulated with 20μM nigericin sodium salt (Sigma-Aldrich) for 30 minutes. Culture supernatants were collected and cells were lysed with M-PER Mammalian Protein Extraction Reagent (Thermo Fisher Scientific) supplemented with 1:100 protease inhibitor mixture (Sigma-Aldrich). Cell lysates and supernatants were subjected to SDS-PAGE analysis and western blotting. The proteins were resuspended in SDS-containing loading buffer, separated on a 15% SDS-PAGE gel, and transferred to nitrocellulose membranes (Amersham Biosciences, Uppsala, Sweden) in transfer buffer (50mM Tris, 40mM glycine, 10% methanol). Membranes were blocked for 1 hour in TBS with 0.1% Tween-20 containing 5% nonfat dry milk and incubated overnight with primary antibodies at 4°C. Primary Abs used included a mouse monoclonal against the p20 subunit of caspase-1 (Adipogen, San Diego, CA, USA), a mouse monoclonal against caspase-11 (Adipogen, San Diego, CA, USA) and a rat monoclonal against GSDMD (Genentech, cell line GN20-13), both at a 1:1000 dilution. Loading control blot was performed using mAb anti–β-actin (Cell Signaling Technology, Danvers, MA) at a 1:1000 dilution. The membranes were washed three times for 5 min in TBS with 0.1% Tween 20 and incubated for 1 h at 25°C with the appropriate HRP-conjugated secondary Ab at a 1:1000 dilution. Immunoreactive bands were visualized using Luminol chemiluminescent HRP substrate (Millipore) and analyzed using the ImageQuant TL Software (GE Healthcare, Buckinghamshire, United Kingdom).

### Confocal microscopy

C57BL/6, *Casp11*^*-/-*^ and *Gsdmd*^*-/-*^ mice were infected with *B*. *abortus* as previously described, and 3 days post-infection they were inoculated i.v. with a single dose of 8μg of Ly-6G PE antibody (clone 1A8; 1:200, BD Bioscience) to each 20g mice. After 2h, spleens were extracted, and whole organ *ex-vivo* confocal microscopy analysis was performed using a Nikon A1 confocal system. Three animals per group were analyzed, and images were taken using a 4x objective for ten random fields per mice. The percentage of red fluorescent pixels was analyzed per organ area per field using ImageJ.

### Increased extracellular [K+] assay and measurement of K^+^

To increased extracellular [K+] assay, BMDMs were seeded at a density of 5 × 10^5^ cells/well in 24-well. We incubated BMDMs in a medium containing 80 mM KCl 1 h before infection. Then, BMDMs were infected with *B*. *abortus* at an MOI of 100 in the same medium for 17 h and IL-1β was measured in the supernatant. Intracellular concentration of K^+^ was determined by fluorescence emission of Asante Potassium Green-2 (APG-2, TEFLabs, Austin, EUA). Briefly, BMDMs (2 × 10^4^) were seeded in black, clear-bottom 96-well plates, infected with *B*. *abortus* at an MOI of 100. After 6 h of infection, cells were incubated with 5 μM APG-2 in RPMI without FBS and phenol red for 30 min. BMDMs were washed with PBS, and the media was replaced with RPMI without phenol red. Four images per well were recorded at 40× magnification with the ImageXpress Micro High-Content Imaging System and processed with MetaXpress High-Content Image Acquisition and Analysis (Molecular Devices). The images were analyzed using ImageJ, and the concentration of intracellular K^+^ in each cell was calculated as a percentage: MFI_540nm_ (inquired cell)/ Σ MFI_540nm_ (control cells) ×100.

### In vivo and in vitro infections

Five mice from each group (C57BL/6, *Casp11*^*-/-*^ and *Gsdmd*^*-/-*^) were infected i.p. with 1 × 10^6^ CFU *B*. *abortus* virulent strain S2308 and sacrificed at 72 h, 1 or 2 weeks postinfection. For *Nlrp3*^*-/-*^, *Casp11*^*-/-*^, *Casp1*^*-/-*^*Casp11*^*Tg*^, *Casp1/11*^*-/-*^, the bacterial load was evaluated at 2 weeks after infection. The spleens were harvested and macerated in 10 ml saline (NaCl 0.9%), serially diluted, and plated in duplicate on *Brucella* Broth agar. After 3 d of incubation at 37°C, the number of CFU was determined as described previously [46]. To measure intracellular multiplication in macrophages, BMDMs were seeded at a density of 5 × 10^5^ cells/well into 24-well tissue culture plates. Cultures were infected at *B*. *abortus* MOI of 10, followed by incubation at 37°C in a 5% CO_2_ atmosphere. For CFU determination, the cultures were lysed in sterile water after 2, 24, and 48 h of infection. Lysates from each well were diluted in water, plated on Brucella broth (BB) agar plates, and incubated for 3 d at 37°C for CFU determination.

### Neutrophil depletion

Neutrophils were depleted by intraperitoneal injection of 100 μg of anti-mouse Ly6G (clone 1A8, BioXcell, West Lebanon, NH, USA) 24 hours before infection i.p. with 1 × 10^6^ CFU *B*. *abortus* virulent strain S2308. The neutrophils depletion was maintained with applications of anti-mouse Ly6G antibodies at intervals of 2 days each dose for 7 days. In these experiments, 100 μg of an isotype control antibody (IgG from rat serum, Sigma-Aldrich, St. Louis, MO, USA) was administered as control. After 1 week of infection, mice were sacrificed, spleens were harvested and CFU counting was performed as described above. Neutrophil depletion was confirmed by flow cytometry analysis of spleen cells from depleted and control mice. The neutrophil population was analyzed by staining 1x10^6^ cells for 30 min on 4°C with fluorescent antibodies against Ly6G (PE, clone 1A8, BD Biosciences). Stained cells were acquired in Attune Flow Cytometer (Applied Biosystems, Waltham, MA, USA) and analyzed using FlowJo software (Tree Star, Ashland, OR, USA).

### Statistical analysis

Statistical analysis was performed using Prism 5.0 software (GraphPad Software, San Diego, CA). The unpaired Student *t* test was used to compare two groups. One-way ANOVA followed by multiple comparisons according to Tukey procedure was used to compare three or more groups. Unless otherwise stated, data are expressed as the mean ± SD. Differences were considered statistically significant at a p value < 0.05.

## Supporting information

S1 Fig Caspase-11 expression in macrophages infected with *B*. *abortus*. BMDMs obtained from C57BL/6, *Casp11*^−/−^, *Casp1*^*−/−*^*Casp11*^*Tg*^ and *Casp1/11*^*−/−*^ mice were primed or not with *E*. *coli* LPS (1 μg/ml) for 4 h and then left uninfected (NI) or stimulated with *B*. *abortus* with MOI of 100 for 17h. Cell lysates were harvested and separated by SDS-PAGE, blotted, and probed with a monoclonal antibody anti–caspase-11 p38/p43 and with rabbit anti-actin polyclonal antibody. NI: uninfected.
(PDF)

S2 Fig Guanylate-binding proteins (GBPs) expression in BMDMs. (A) BMDMs from C57BL/6 mice were transfected with LPS (5 μg/ml) from *B*. *abortus* using FugeneHD. After 17h of infection, RNA was extracted, purified, and qPCR was performed to measure GBP1, GBP2, GBP3, GBP5 and GBP7 expression levels. * p<0.05 when compared to Fugene HD alone. (B) BMDMs from C57BL/6 mice were transfected with siRNA from siGENOME SMARTpools (Dharmacon) for siRNA control, GBP1, GBP3, GBP5 or GBP7 for 46h and then transfected with LPS (5 μg/ml) from *B*. *abortus*. After 17h, total RNA was extracted, purified and qPCR performed to measure GBP1, GBP3, GBP5 and GBP7 expression levels. * p<0.05 when compared to siRNA control.
(PDF)

S3 Fig Caspase-11 and GSDMD are dispensable for the restriction of *B*. *abortus* replication in macrophages. BMDMs obtained from C57BL/6, *Casp11*^*−/−*^ and *Gsdmd*^*−/−*^ mice were infected with *B*. *abortus* in a MOI of 100 per well. Cultures were incubated for 2, 24 and 48 h for CFU determination. Shown are the averages ± SD from triplicate wells.
(PDF)

S4 Fig Caspase-11 but not caspase-1 and NLRP3 is required to host control of *B*. *abortus* infection in mice. C57BL/6, *Casp11*^−/−^, *Casp1*^*−/−*^*Casp11*^*Tg*^, *Nlrp3*^*−/−*^ and *Casp1/11*^*−/−*^ mice were infected intraperitoneally with 1x10^6^ CFU of *B*. *abortus*. Mice were sacrificed 2 weeks postinfection and diluted spleen homogenates were added to BB medium agar plates for CFU determination. Data are the mean ± SD of five mice/group. Statistically significant differences of *Casp11*^*−/−*^ and *Casp1/11*^*−/−*^ compared to wild-type mice are denoted by an asterisk, *p < 0.05. The graph is representative of three independent experiments.
(PDF)

S5 Fig KC production in response to *B*. *abortus* infection in mouse spleens. C57BL/6, *Casp11*^*-/-*^ and *Gsdmd*^*-/-*^ mice were infected intraperitoneally with 1 x 10^6^ CFU of *B*. *abortus*. Mice were sacrificed 2 weeks postinfection, spleens were collected and processed to extract the cytokine. The concentration of KC in spleen homogenates were measured by ELISA. Data show the mean ± SD of five mice/group. *p < 0.05 compared to wild-type mice.
(PDF)

S6 Fig The anti-Ly6g antibody treatment efficiently depleted neutrophils in *B*. *abortus* infected mice. WT mice received Ly6G-depleting antibody (100 μg/animal) every 2 days during seven days. Neutrophil depletion in the spleen was measured by flow cytometry. n = 5 per group per experiment. FACS plots are representative of 2 independent experiments.
(PDF)

S7 Fig IL-1α-deficient mice did not show increased bacterial load after *B*. *abortus* infection when compared to wild-type animals. C57BL/6 and *IL-1α*^*−/−*^ mice were infected intraperitoneally with 1x10^6^ CFU of *B*. *abortus*. Mice were sacrificed 2 weeks postinfection and diluted spleen homogenates were added to BB medium agar plates for CFU determination. Data are the mean ± SD of five mice/group. The graph is representative of two independent experiments.
(PDF)
