# Supplementary figures and images for: Guanylate-binding protein 5 licenses caspase-11 for Gasdermin-D mediated host resistance to Brucella abortus infection
Source: PLoS Pathog. 2018 Dec 27;14(12):e1007519. doi: 10.1371/journal.ppat.1007519 (PMC6326519; doi:10.1371/journal.ppat.1007519)

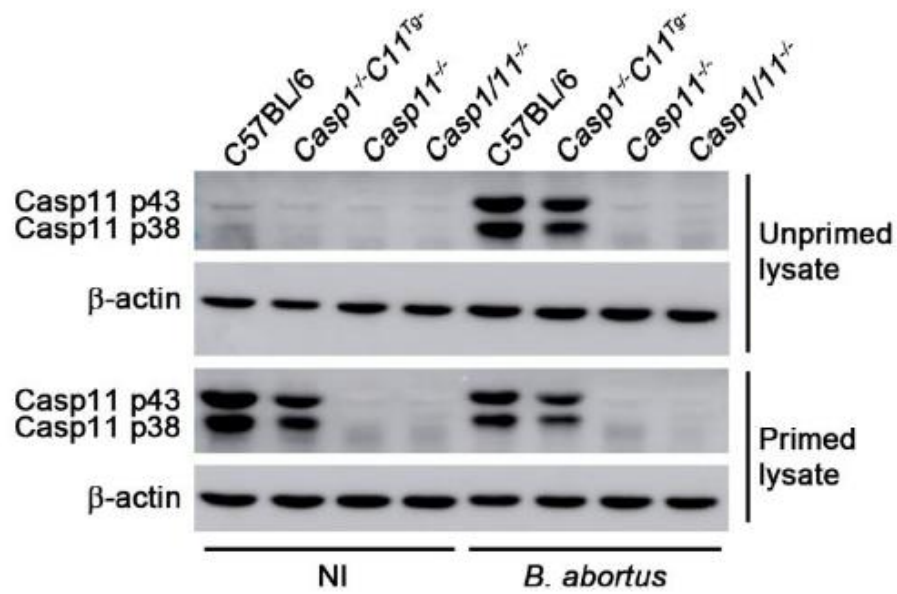

**S1 Fig. Caspase-11 expression in macrophages infected with *B. abortus*.**

Supplement: S1 Fig — BMDMs obtained from C57BL/6, Casp11−/−, Casp1−/−Casp11Tg and Casp1/11−/− mice were primed or not with E. coli LPS (1 μg/ml) for 4 h and then left uninfected (NI) or stimulated with B. abortus with MOI of 100 for 17h. Cell lysates were harvested and separated by SDS-PAGE, blotted, and probed with a monoclonal antibody anti–caspase-11 p38/p43 and with rabbit anti-actin polyclonal antibody. NI: uninfected. (PDF) [file ppat.1007519.s001.pdf]

**A**

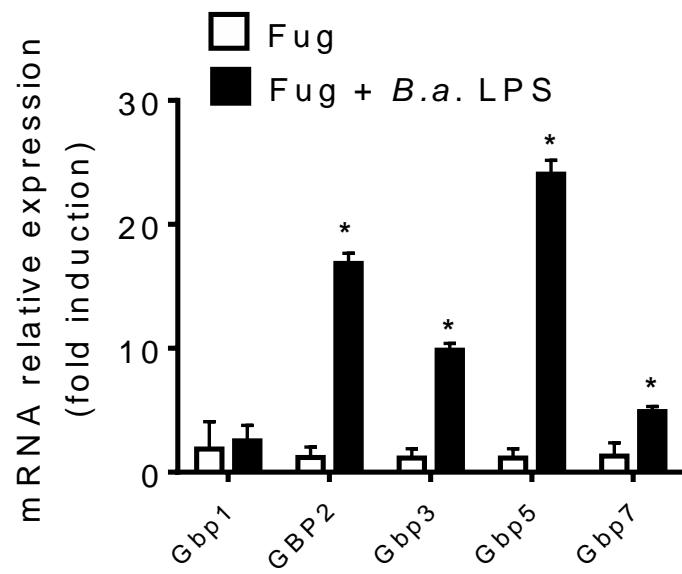

**B**

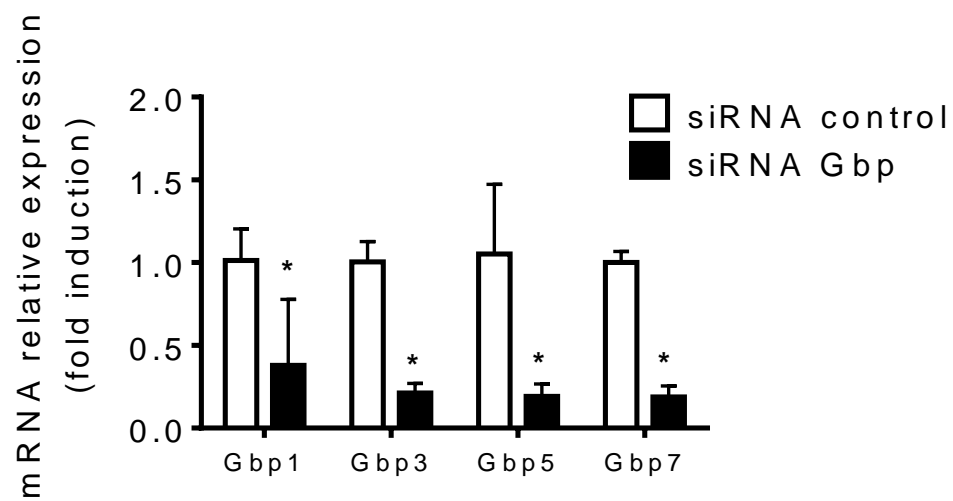

**S2 Fig. Guanylate-binding proteins (GBPs) expression in BMDMs.**

Supplement: S2 Fig — (A) BMDMs from C57BL/6 mice were transfected with LPS (5 μg/ml) from B. abortus using FugeneHD. After 17h of infection, RNA was extracted, purified, and qPCR was performed to measure GBP1, GBP2, GBP3, GBP5 and GBP7 expression levels. * p<0.05 when compared to Fugene HD alone. (B) BMDMs from C57BL/6 mice were transfected with siRNA from siGENOME SMARTpools (Dharmacon) for siRNA control, GBP1, GBP3, GBP5 or GBP7 for 46h and then transfected with LPS (5 μg/ml) from B. abortus. After 17h, total RNA was extracted, purified and qPCR performed to measure GBP1, GBP3, GBP5 and GBP7 expression levels. * p<0.05 when compared to siRNA control. (PDF) [file ppat.1007519.s002.pdf]

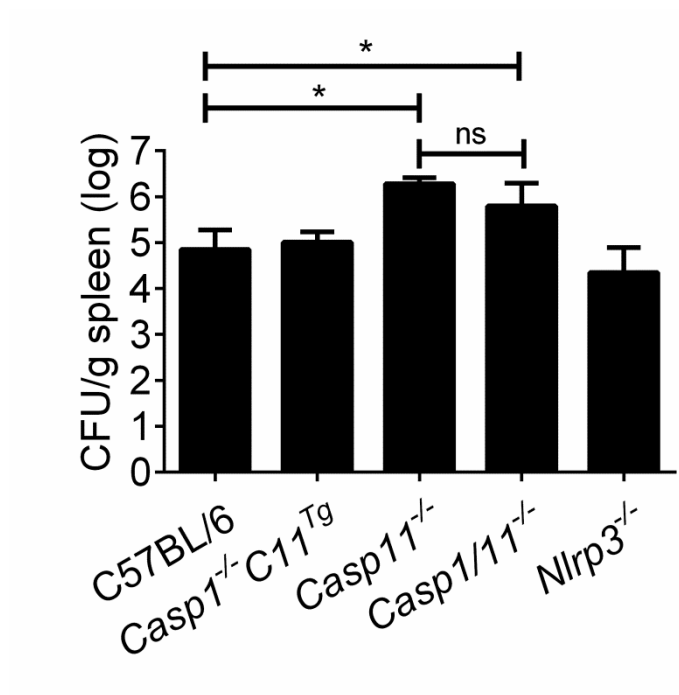

**S4 Fig. Caspase-11 but not caspase-1 and NLRP3 is required to host control of *B. abortus* infection in mice.**

Supplement: S4 Fig — C57BL/6, Casp11−/−, Casp1−/−Casp11Tg, Nlrp3−/− and Casp1/11−/− mice were infected intraperitoneally with 1x10^6^ CFU of B. abortus. Mice were sacrificed 2 weeks postinfection and diluted spleen homogenates were added to BB medium agar plates for CFU determination. Data are the mean ± SD of five mice/group. Statistically significant differences of Casp11−/− and Casp1/11−/− compared to wild-type mice are denoted by an asterisk, *p < 0.05. The graph is representative of three independent experiments. (PDF) [file ppat.1007519.s004.pdf]

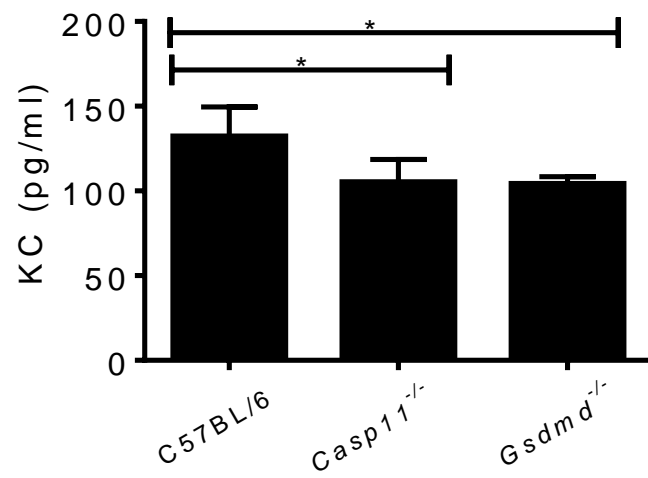

**S5 Fig. KC production in response to *B. abortus* infection in mouse spleens.**

Supplement: S5 Fig — C57BL/6, Casp11-/- and Gsdmd-/- mice were infected intraperitoneally with 1 x 10^6^ CFU of B. abortus. Mice were sacrificed 2 weeks postinfection, spleens were collected and processed to extract the cytokine. The concentration of KC in spleen homogenates were measured by ELISA. Data show the mean ± SD of five mice/group. *p < 0.05 compared to wild-type mice. (PDF) [file ppat.1007519.s005.pdf]
