# Supplementary material for: Guanylate-binding protein 5 licenses caspase-11 for Gasdermin-D mediated host resistance to Brucella abortus infection
Source: PLoS Pathog. 2018 Dec 27;14(12):e1007519. doi: 10.1371/journal.ppat.1007519 (PMC6326519; doi:10.1371/journal.ppat.1007519)
Supplement: S3 Fig — BMDMs obtained from C57BL/6, Casp11−/− and Gsdmd−/− mice were infected with B. abortus in a MOI of 100 per well. Cultures were incubated for 2, 24 and 48 h for CFU determination. Shown are the averages ± SD from triplicate wells. (PDF) [file ppat.1007519.s003.pdf]

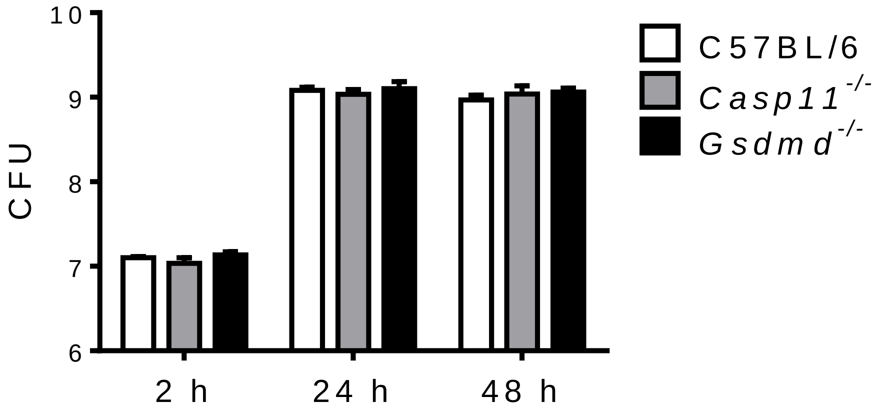

S3 Fig. Caspase-11 and GSDMD are dispensable for the restriction of *B. abortus* replication in macrophages.
