# Supplementary material for: Guanylate-binding protein 5 licenses caspase-11 for Gasdermin-D mediated host resistance to Brucella abortus infection
Source: PLoS Pathog. 2018 Dec 27;14(12):e1007519. doi: 10.1371/journal.ppat.1007519 (PMC6326519; doi:10.1371/journal.ppat.1007519)
Supplement: S6 Fig — WT mice received Ly6G-depleting antibody (100 μg/animal) every 2 days during seven days. Neutrophil depletion in the spleen was measured by flow cytometry. n = 5 per group per experiment. FACS plots are representative of 2 independent experiments. (PDF) [file ppat.1007519.s006.pdf]

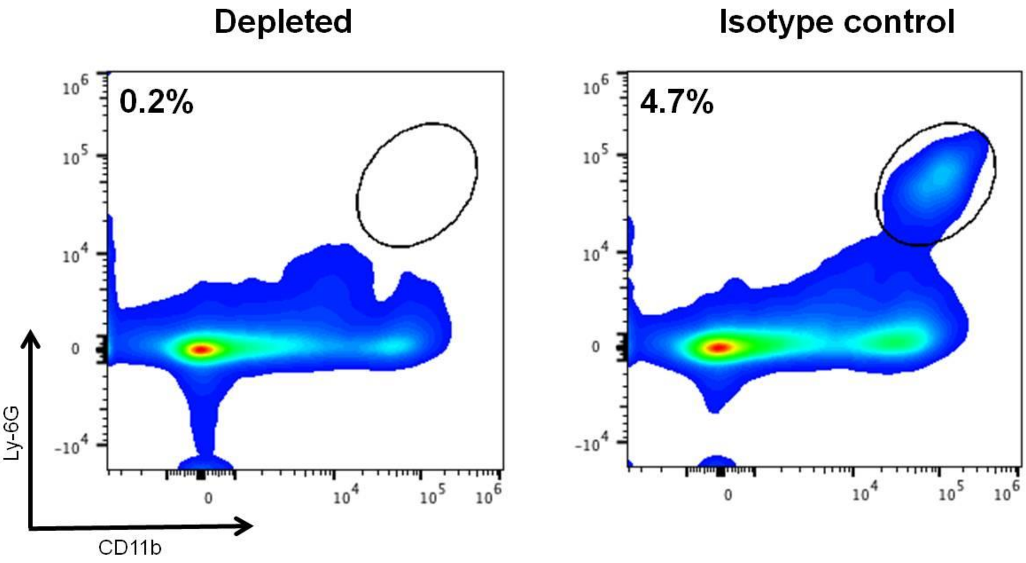

S6 Fig. The anti-Ly6g antibody treatment efficiently depleted neutrophils in *B. abortus* infected mice.
