# Supplementary material for: Guanylate-binding protein 5 licenses caspase-11 for Gasdermin-D mediated host resistance to Brucella abortus infection
Source: PLoS Pathog. 2018 Dec 27;14(12):e1007519. doi: 10.1371/journal.ppat.1007519 (PMC6326519; doi:10.1371/journal.ppat.1007519)
Supplement: S7 Fig — C57BL/6 and IL-1α−/− mice were infected intraperitoneally with 1x10^6^ CFU of B. abortus. Mice were sacrificed 2 weeks postinfection and diluted spleen homogenates were added to BB medium agar plates for CFU determination. Data are the mean ± SD of five mice/group. The graph is representative of two independent experiments. (PDF) [file ppat.1007519.s007.pdf]

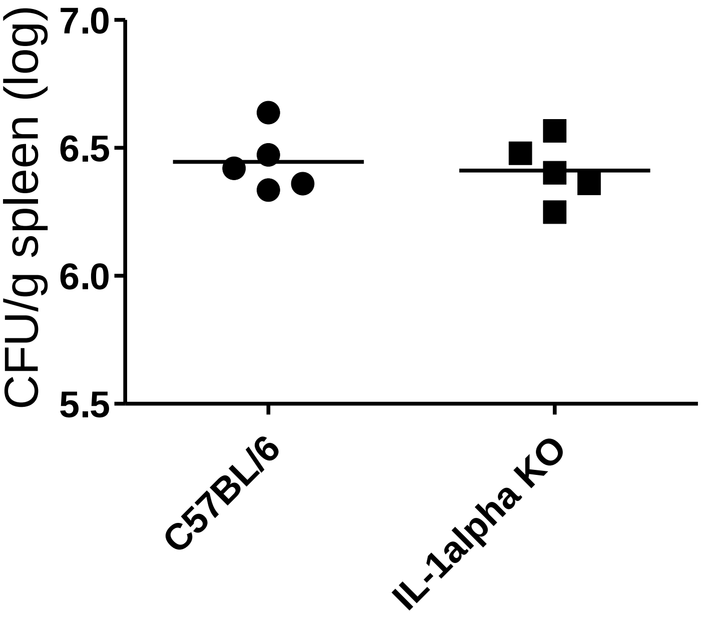

S7 Fig. IL-1α-deficient mice did not show increased bacterial load after *B. abortus* infection when compared to wild-type animals.
